# Reverse-engineering the *Arabidopsis thaliana *transcriptional network under changing environmental conditions

**DOI:** 10.1186/gb-2009-10-9-r96

**Published:** 2009-09-15

**Authors:** Javier Carrera, Guillermo Rodrigo, Alfonso Jaramillo, Santiago F Elena

**Affiliations:** 1 Instituto de Biología Molecular y Celular de Plantas, Consejo Superior de Investigaciones Científicas-UPV, Ingeniero Fausto Elio s/n, 46022 València, Spain; 2 ITACA, Universidad Politécnica de Valencia, Ingeniero Fausto Elio s/n, 46022 València, Spain; 3 Laboratoire de Biochimie, École-Polytechnique-CNRS UMR7654, Route de Saclay, 91128 Palaiseau, France; 4 Epigenomics Project, Genopole-Université d'Évry Val d'Essonne-CNRS UPS3201, 523 Terrasses de l'Agora, 91034 Évry, France; 5 The Santa Fe Institute, Hyde Park Road, Santa Fe, NM 87501, USA

## Abstract

**Background:**

Understanding the molecular mechanisms plants have evolved to adapt their biological activities to a constantly changing environment is an intriguing question and one that requires a systems biology approach. Here we present a network analysis of genome-wide expression data combined with reverse-engineering network modeling to dissect the transcriptional control of *Arabidopsis thaliana*. The regulatory network is inferred by using an assembly of microarray data containing steady-state RNA expression levels from several growth conditions, developmental stages, biotic and abiotic stresses, and a variety of mutant genotypes.

**Results:**

We show that the *A. thaliana *regulatory network has the characteristic properties of hierarchical networks. We successfully applied our quantitative network model to predict the full transcriptome of the plant for a set of microarray experiments not included in the training dataset. We also used our model to analyze the robustness in expression levels conferred by network motifs such as the coherent feed-forward loop. In addition, the meta-analysis presented here has allowed us to identify regulatory and robust genetic structures.

**Conclusions:**

These data suggest that *A. thaliana *has evolved high connectivity in terms of transcriptional regulation among cellular functions involved in response and adaptation to changing environments, while gene networks constitutively expressed or less related to stress response are characterized by a lower connectivity. Taken together, these findings suggest conserved regulatory strategies that have been selected during the evolutionary history of this eukaryote.

## Abstract 2

An Arabidopsis thaliana transcriptional network reveals regulatory mechanisms for the control of genes related to stress adaptation.

## Background

Living organisms have evolved molecular circuitries with the aim of promoting their own development under dynamically changing environments. In particular, plants are not able to evade those changes and have had to evolve robust methods to cope with environmental stress and recovery mechanisms. Genomic sequences specify the context-dependent gene expression programs to render cells, tissues, organs and, finally, organisms. Then, at any moment during the cell cycle and at each stage of an organism's development, and in response to environmental conditions, each cell is the product of specific and well defined programs involving the coordinated transcription of thousands of genes. Thus, the elucidation of such programs in terms of the regulatory interactions involved is pivotal for the understanding of how organisms have evolved and what environments may have conditioned evolutionary trajectories the most. However, we still have little understanding of how this highly tuned process is achieved for most organisms, and the surface of the problem is only just being scratched for a handful of model organisms, such as the bacterium *Escherichia coli *[1], the yeast *Saccharomyces cerevisiae *[2], the nematode *Caenorhabditis elegans *[3], the plant *Arabidopsis thaliana *[4,5], and, to a lesser extent, humans [6].

Meta-analyses of microarray data collections may now be used to construct biological networks that systematically categorize all molecules and describe their functions and interactions. Networks can integrate biological functions of cells, organs, and organisms. During recent years, there has been a tremendous effort in the development and improvement of techniques to infer gene connectivity. Clustering approaches [7-11] and information theory methods [12-16] have been used to infer regulatory networks. Bayesian methods [17-20] can give accurate networks with low coverage but at a high computational cost.

The analysis of the expression of the *A. thaliana *transcriptome offers the potential to identify prevailing cellular processes, to associate genes with particular biological functions, and to assign otherwise unknown genes to biological responses. Previous attempts to model the *A. thaliana *gene network used methods such as fuzzy *k-*means clustering [21], graphical Gaussian models [4], and Markov chain graph clustering [5,15]. The inconvenience of the first approach is that clustering describes genes based on a characteristic property common to all genes, but it is difficult to deduce a pathway structure from this property alone because pathways would have to be concerned with co-expression features that transcend such cluster structure. The second approach assumes that the number of microarray slides should be much larger than the number of genes analyzed or approximations must be taken (for example, empirical Bayes with bootstrap re-sampling or shrinkage approaches). The last approach is based on Person's correlations and, therefore, strongly sensitive to outliers and to violations of the implicit assumption of linear relationships among genes. In this article, we present a predictable genome model from a regulatory scaffold inferred by using probabilistic methods [15] and estimate the corresponding kinetic parameters using linear regression [22-25]. We analyze the topological properties and predictive power of the inferred regulatory model. We evaluate the performance of the network by predicting already known transcriptional regulations and assess the functional relevance and reproducibility of the co-expression patterns detected. Finally, we discuss the evolutionary implications of transcriptional control in plants.

## Results

High-throughput technologies combined with rigorous and biologically rooted modeling will allow understanding of how simple genetic or environmental perturbations influence the dynamic behavior of cellular genetic and metabolic networks [26]. However, transcriptomic data need to be properly integrated to formulate a model that can be used for making quantitative predictions on how the environment interacts with cellular networks to affect phenotypic responses. At the end, the accurate prediction of this quantitative behavior will open the possibility of re-engineering cellular circuits. To reach this end, we have attempted the integration of experimental and computational approaches to construct a predictive gene regulatory network model covering the full transcriptome of the model plant *A. thaliana*.

### Genome-wide transcriptional control in *A. thaliana*

**Figure 1:**
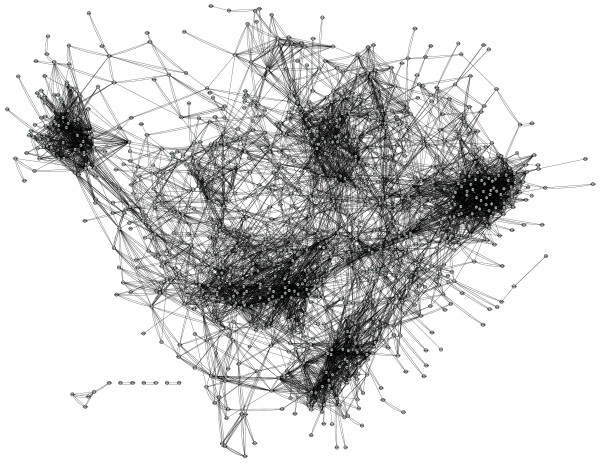
Plot of the inferred regulatory network of *A. thaliana *visualized using Cytoscape. Nodes only represent TFs.

In the present work, we have applied a recently developed inference methodology, InferGene [25], to obtain a gene regulatory model suitable for analyzing optimality and allowing study of the transcriptional control response under changing environments in *A. thaliana*. For this, we have considered the Affymetrix chip for the *A. thaliana *genome, from which we selected 22,094 non-redundant genes, of which about 1,187 are putative transcription factors (TFs; see Materials and methods). The data used for the inference procedure were a compendium of 1,436 Affymetrix microarray hybridization experiments publicly available at The *Arabidopsis *Information Resource (TAIR) website; these were normalized using the robust multi-array average method [27]. Here we used the whole expression set (1,436 experiments) to construct the model. In Figure 1 we show the inferred transcriptional regulatory network of *A. thaliana *drawn using the Cytoscape viewer [28]; Table 1 collates some parameters describing the topology of the network.

**Table 1:** Topological parameters of the inferred transcription network of *A. thaliana*

**Parameter**	**Value**
Clustering coefficient	0.319
Network diameter	13
Characteristic path length	5.065
Number of connected genes	18,169
Number of regulations inferred	128,422
Network density	7.78 × 10^-4^
Constitutive genes	3,952 (17.89%)
Genes regulated by one TF	3,111 (14.08%)
Genes regulated by two TFs	2,352 (10.64%)
Genes regulated by three TFs	1,966 (8.90%)
Genes regulated by four TFs	1,606 (7.27%)
Genes regulated by five TFs	1,393 (6.30%)
Genes regulated by more than five TFs	7,714 (34.91%)

Three types of efficiencies, precision (*P*), sensitivity (*S*) and absolute efficiency (*F*), have been computed to assess the ability of the above inferred network to predict the 448 experimentally validated transcriptional regulations collected in the AtRegNet database. *P *is the fraction of predicted interactions that are correct:



and *S *the fraction of all known interactions that are discovered by the model:



where *TP *is the number of true positives, *FN *the number of false negatives and *FP *the number of false positives. *F *thus represents the absolute efficiency and it is computed as:



which is the harmonic mean of precision and sensitivity. Indeed, precision and sensitivity are necessarily negatively correlated performance statistics, and these two values were set up so they maximize global performance (*F*) by selecting values > 5 (Figures S1 and S2 in Additional data file 1) for the *z*-score used as threshold to predict the transcriptional regulations. Figure S3 in Additional data file 1 shows *P*, *S *and *F *as a function of the *z*-score threshold. Sensitivity is maximized (*S *= 100%) for *z *= 0 (that is, a high number of regulations but very low confidence) while precision is maximized (*P *= 100%) for *z *= 11 (that is, high confidence but a very low number of regulations). The optimum value is reached for *z *= 5, a value for which *F *= 26% (*P *= 40% and *S *= 20%). In a recent study, a smaller network topology has been proposed for *A. thaliana *[4]. This network contains 18,625 regulations and an *F *= 3.7% (*P *= 88% but *S *= 1.8%), relative to the AtRegNet reference dataset.

InferGene predicts that more than half of the genes are controlled by constitutive promoters (17.89%) or by promoters regulated by less than three TFs (Table 1). Also, from a purely topological perspective, the inferred transcriptional network of *A. thaliana *is weakly connected directed, containing 18,169 connected genes (Table 1), while the size of the largest strongly connected component contains only 730 nodes, all of which are TFs. In addition, it has a high density (0.078%; Table 1); this parameter is the normalized average connectivity of a gene in the network in comparison to values reported in similar studies on other organisms. For example, Lee *et al*. [2] suggested a network density of 0.0027% for *S. cerevisiae*, while we previously reported a value of 0.036% for the inferred network for *E. coli *[25]. The characteristic path length [29] of the network follows a Gaussian distribution, with an average value of 5.065 edges (Table 1; Figure S4 in Additional data file 1) and, specifically, the distance between two genes for which a path exists ranges from 1 to 13 edges. In a previous study, we estimated that the characteristic path length for the *E. coli *network was 1 [25], much smaller than that for *A. thaliana*. Furthermore, the *E. coli *inferred network did not contain any strongly connected components and its largest weakly directed subnetwork contained only four TFs.

**Figure 2:**
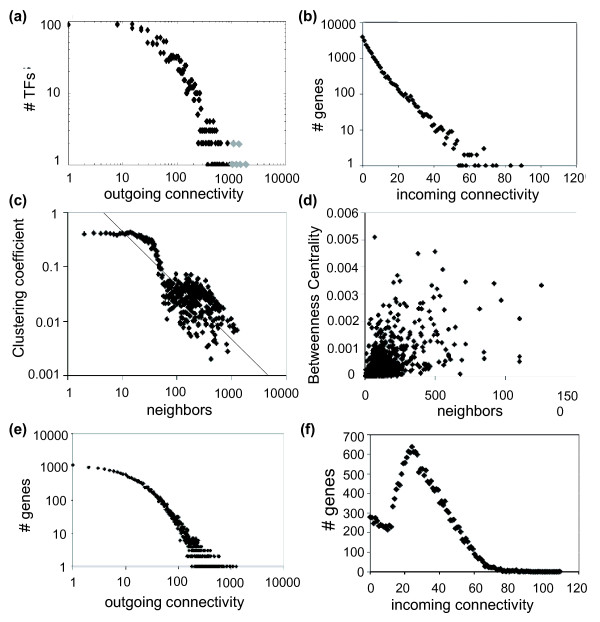
Analyses of the regulatory network of *A. thaliana*. Distributions for the transcriptional network of: **(a) **outgoing connectivity showing the master regulators from Table 2 in gray; **(b) **incoming connectivity; **(c) **clustering coefficient; and **(d) **betweenness centrality. Distributions for the non-transcriptional network of: **(e) **outgoing connectivity; and **(f) **incoming connectivity.

Other relevant statistical properties of networks are the stress distribution (Figure S5 in Additional data file 1) - that is, the number of paths in which a gene is involved - and the betweenness centrality distribution (Figure 2d) - that is, the number of shortest pathways in which a particular gene is involved. Both distributions are highly asymmetrical, with many nodes having low betweenness centrality and only a few cases with high betweenness centrality (Figure 2d), and with the number of shortest paths per gene smoothly increasing until reaching a maximum of approximately 10^5 ^short paths per gene followed by a drastic drop, with very few genes (around 5) having 10^7 ^short paths (Figure S5 in Additional data file 1). Ten genes (*At1g32330*, *At4g26930*, *At1g24110*, *At4g24490*, *At2g36590*, *At1g01030*, *At1g76900*, *At2g19050*, *At2g03840*, and *At3g19870*) are connected among themselves but remain isolated from the rest of the main network (Figure 1); the number of shortest paths for these genes ranges from 1 to 3 (Figure S5 in Additional data file 1). All these genes but the last are involved in several and apparently loosely related Gene Ontology (GO) functional categories that include regulation of transcription, transportation and signal transduction, and development and senescence.

Next, we sought to explore whether the inferred regulatory network has scale-free properties. It has been suggested that the distribution of outgoing connections should belong to the class of scale-free small-world networks, representing the potential of TFs to regulate multiple target genes, whereas the distribution of incoming connectivities would be more exponential-like because regulation by multiple TFs should be less common than regulation of several targets by a given TF [30]. Figure 2a shows the distribution of outgoing connectivities per TF, whereas Figure 2b shows the same distribution but only for incoming connectivities per gene. As expected, the outgoing connectivity is best fitted by a truncated power-law (that is, the Weibull distribution) with exponent *γ *= 0.902 and cutoff *k*_*c *_= 99.093 (Table S1 in Additional data file 2; *R*^2 ^= 0.949; Akaike's weight over a set of 10 competing models > 99.99%). This distribution indicates that outgoing connectivity has a scale-free behavior in the range 1 ≤ *k *<*k*_*c *_but deviates from this for connectivities over the cutoff. According to Barabási and Oltvai [31], scale-free properties arise when hub genes are related in a hierarchical way, with the hub receiving most links being connected to a small fraction of all nodes. In the case of incoming connectivity, the model that better describes the data is a restricted exponential, the half-normal distribution (Table S1 in Additional data file 2; *R*^2 ^= 0.983; Akaike's weight > 99.99%). Taken together, these two observations suggest that the *A. thaliana *transcriptional network contains a few highly connected regulators (Table 2) that play a central role in mediating interactions among a large number of less connected genes. Notice that 88.4% of the TFs regulate more than 10 genes, 36.3% regulate more than 100 genes and just 2.6% control over 500 genes. For the sake of comparison, it is worth mentioning that, in the case of *S. cerevisiae*, the critical exponents estimated for the outgoing connectivity distribution (*γ *= 0.96 [2,32]) are quite similar to that reported here. However, the estimate obtained for *E. coli *was smaller (*γ *= 0.87), a result that suggests that hubs are more important in bacteria than in the two eukaryotes [31].

**Table 2:** The ten transcription factors with the most regulatory effects (highest outgoing connectivity)

**Transcription factor**	**Outgoing connectivity**	**Gene annotation**	**GO pathways (level 5)**
*At4g17695*	1254	*KAN3 *(*KANDI 3*)	Transcription; regulation of cellular metabolic process
*At1g77200*	1103	*AP2*	Transcription; regulation of cellular metabolic process; RNA metabolic process
*At2g17040*	1100	*ANAC036 *(*Arabidopsis *NAC domain containing protein 36)	Transcription; regulation of cellular metabolic process; RNA metabolic process
*At5g16560*	1100	*KAN*	Reproductive structure development; regionalization; organ development; cell fate commitment
*At2g47900*	971	*AtTLP3 *(tubby like protein 3)	Transcription; regulation of cellular metabolic process
*At2g28700*	921	*AGL46*	Transcription; regulation of cellular metabolic process; RNA metabolic process
*At5g07690*	850	*MYB29 *(myb domain protein 29)	Transcription; response to gibberellin stimulus; regulation of cellular metabolic process; RNA metabolic process
*At4g14920*	846	PHD finger	Transcription; regulation of cellular metabolic process; RNA metabolic process
*At3g23240*	816	*ATERF1*/*ERF1 *(ethylene response factor 1)	Response to ethylene stimulus; transcription; regulation of cellular metabolic process; intracellular signaling cascade; two-component signal transduction system; RNA metabolic process
*At3g30210*	721	*MYB121 *(myb domain protein 121)	Response to abscisic acid stimulus; transcription; regulation of cellular metabolic process; RNA metabolic process

We have validated the set of predicted targets for the 25% most highly connected TFs using AtRegNet, recovering 80% of known interactions for the regulatory model and up to 85% for the effective model (that is, the one containing both gene-gene and gene-TF interactions). Figure 2c shows that the scaling of the average clustering coefficient with the number of genes with *k*-connections is approximately linear in a log-log scale in the range 1 to 10,000 for neighbors with slope -1.05 (*R*^2 ^= 0.850). Barabási and Oltvai [31] and Ravasz and Barabási [33] have suggested that whenever clustering scales with the number of nodes with slope -1, as in our case, it has to be taken as a strong indication of hierarchical modularity - that is, genes cluster in higher-order units of different modularity - a finding that has been suggested as general for system-level cellular organization in plants [34]. Similarly, when the effective model is analyzed, it shows similar results to those for the regulatory model. The outgoing connectivity per gene follows a truncated power law with scale-free behavior up to *k*_*c *_= 21.341 connections per gene and with an exponent *γ *= 0.765 (Table S1 in Additional data file 2; *R*^2 ^= 0.998, Akaike's weight > 99.99%; Figure 2e). Figure 2f shows that the incoming connectivity per gene does not present scale-free properties as it fits to a normal distribution (Table S1 in Additional data file 2; *R*^2 ^= 0.998, Akaike's weight > 99.99%).

**Figure 3:**
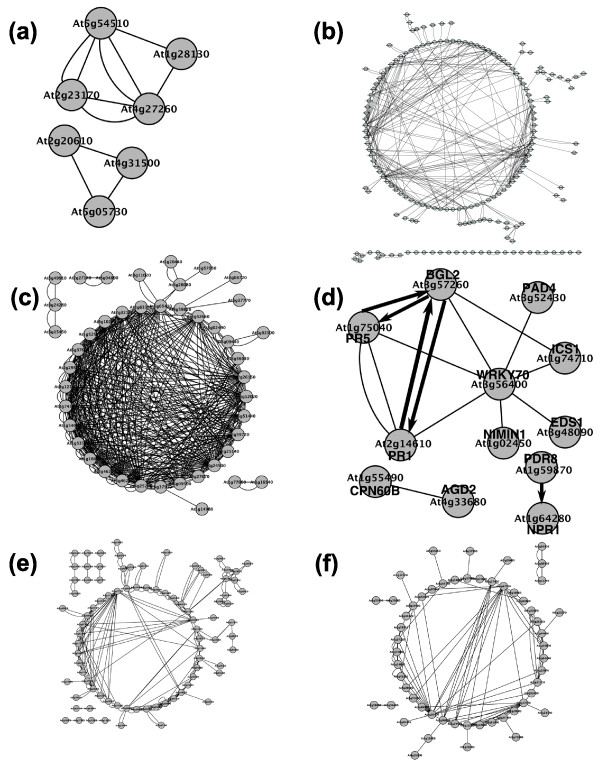
Transcriptional subnetworks with high clustering coefficients corresponding to the following GO pathways: **(a) **auxin metabolic process; **(b) **response to other organism; **(c) **response to heat; **(d) **systemic acquired resistance (experimentally verified regulations are represented with thick edges); **(e) **response to salt stress; and **(f) **immune response.

The environment significantly influences the dynamic expression and assembly of all components encoded in the *A. thaliana *genome into functional biological subnetworks. We have computed the clustering coefficient for all subnetworks with the largest normalized index of connectivity between genes involved in the subnetwork. The subnetworks were then ranked according to these numbers and the top 12 networks are shown in Table 3. Interestingly, four of these highly connected subnetworks are involved in responses to external influences - for example, responses to pathogens and other processes related to abiotic stresses (heat, salinity, light, reduction/oxidation). For the sake of illustration, Figure 3 shows the inferred subnetworks for three abiotic and three biotic responses. In particular, we have made a comprehensive analysis for the subnetwork of systemic acquired resistance (Figure 3d) and found that the fraction of predicted interactions is *P *= 33%. Not surprisingly, all genes involved in this subnetwork are associated with GO categories related to responses to stress, such as defense against pathogens, responses to other organisms such as fungi, bacteria and insects, and responses to cold.

**Table 3:** Clustering coefficient of different Gene Ontology pathways in *A. thaliana*

**GO pathways**	Clustering coefficient*	**Number of connected genes**	**Number of genes**
Auxin metabolic process	0.643	7	31
Response to heat	0.455	44	93
Hydrogen transport	0.335	20	54
Gravitropism	0.250	8	24
Alcohol biosynthetic process	0.233	5	18
Response to salt stress	0.204	87	148
Systemic acquired resistance	0.201	12	21
Immune response	0.190	55	112
Cell morphogenesis	0.153	72	156
Response to other organism	0.105	92	147
Response to bacterium	0.099	34	87
Response to light stimulus	0.088	138	246

*The clustering coefficient for the random subnetworks is 0.005, as computed from 10 subsets of 100 genes each.

### Transcriptomic profile prediction

The basic premise of our approach is to use transcriptomic data from multiple perturbation experiments (either genetic or environmental) and quantitatively measure steady-state RNA concentrations to assimilate these expression profiles into a network model that can recapitulate all observations. We also developed a test model that excludes 10% of experiments to quantify prediction power. This dataset was randomly split into two subsets. The first, larger subset contained 1,292 experiments and was used as a training set for inferring a transcription network containing 128,422 regulatory interactions. The second, smaller subset contained 144 array experiments and was used for validation purposes.

**Figure 4:**
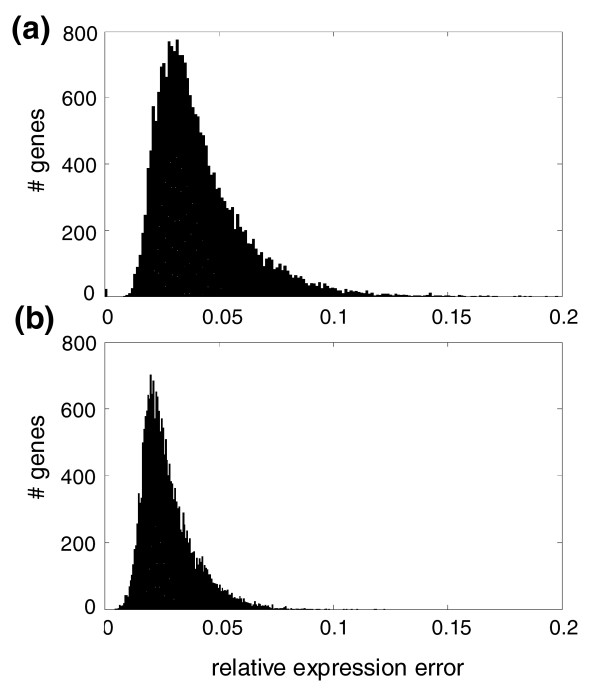
Histogram of the relative gene expression error in **(a)** the transcriptional test model (with an average error of 0.0402) and **(b)** the effective model (with an average error of 0.0280). Errors were obtained from the comparison of the predicted model obtained from the training dataset and the experimental determinations contained in the random test dataset.

As a first measure of the performance of our test model network in predicting responses to stresses, we used it along with the expression levels of all the TFs for each experimental condition, *c*, to predict global expression profiles. Then, the predicted expression values for each of the 22,094 individual genes included in the Affymetrix array, , were compared with the corresponding empirical measurements, *y*_*gc*_, using the deviation statistic:



where *N*_*c *_= 144 is the number of microarray experiments included in the random tester dataset. Figure 4a shows the distribution of Δ_*g *_for all genes included in the predicted *A. thaliana *transcriptional network. The distribution of errors has a median value of 3.66% and is significantly asymmetrical (skewness 1.709 ± 0.017, *P *< 0.0001), with most genes having a relatively low error but with some genes whose expression is estimated having errors > 10% and in a few instances even > 16%. How does this predictive performance compare to that obtained for other organisms, for example, *E. coli*? In a previous study, we constructed a transcriptional network containing 4,345 genes and 328 TFs from *E. coli *[25] using a dataset containing 189 experimental conditions. For this network, the average error over the training set was similar (3.68%) to the values reported above but with the error distribution being even more asymmetrical (skewness 2.314 ± 0.017, *P *< 0.0001). The average error over the *E. coli *test set (4.80%) was larger. Figure 4b shows the distribution of Δ_*g *_for gene-gene and gene-TF interactions, which is also significantly asymmetrical (skewness 1.455 ± 0.017, *P *< 0.001), although in this case the median error is reduced to 2.71% and, in all cases, the error was < 9%. Both distributions significantly differ in shape (Kolmogorov-Smirnov test *P *< 0.001) and location (Mann-Whitney test *P *< 0.001), with the latter being narrower and centered around a lower expression error.

One may ask whether the predictability of our model was driven by TFs and not by non-TF genes. To test this possibility, we proceeded as follows. First, we selected a random set of 1,187 non-TF genes and used them to construct the corresponding pseudo-transcriptional network. Then we evaluated its performance as described above. The level of precision reached was undistinguishable from that of the previous model, with the distribution of relative expression error obtained fully overlapping with thar shown in Figure 4b (data not shown). We conclude from this analysis that TFs do not have stronger predictive power than other genes. This could be rationalized because, in terms of mathematical equations, genes that are coexpressed with the TFs have *a priori *equal chances to work as regulatory elements. On the other hand, we have also constructed an effective model excluding the TFs from the set of predictors and observed that the relative expression error decreased proportionally to the number of excluded TFs.

As a second step for the predictability of our test model, we computed Pearson correlation coefficients (*r*) between the experimental and predicted gene expression levels for all microarray experiments and observed that, as expected, genes having high *r *also have low Δ_*g *_(Figure S6 in Additional data file 1). In addition, we noticed that the predictability of the expression of those genes with high *r *depends on a reduced set of TFs (Figure S7a in Additional data file 1 shows that the critical mass of points concentrates in a region with high *r *and a low number of predictors), suggesting that a selective pressure exists to introduce indirect regulations as a way to increase robustness of genetic systems to dynamic environments. Figure S7a in Additional data file 1 also shows that the model does not tend to add large numbers of regulations as a way to minimize expression error and, by contrast, the highest density of values corresponds to a rather low number of regulations (between 0 and 30). The average incoming connectivity values estimated for *E. coli *[25] and *S. cerevisiae *[2] were 1.56 and 2.26 regulators, respectively. The comparison of these figures with the data reported here suggests that *r *does not significantly increase beyond a given number of regulations.

Nonetheless, a few genes were predicted to have more than 60 regulations. Looking at just the 20 most extremely regulated genes in Figure S7a in Additional data file 1, the results are interesting: the two most extreme cases correspond, respectively, with gypsy- and copia-like retrotransposons (89 and 83 connections to TFs, respectively), nine genes are annotated as unknown proteins, two are annotated as belonging to the F-box family but without any assigned biological process, one has been assigned as a putative protein kinase, five have been loosely assigned to transcription, translation, transport and secondary metabolism, and the only one with a well defined function is the *At2g26330 *locus, which encodes the ERECTA receptor of protein kinases involved in several developmental roles as well as in response to bacterial infections. Moreover, Figure S7b, c in Additional data file 1 shows a histogram of *r *per gene over 1,292 experiments in the training set and 144 conditions in the test set, respectively. The average *r *for the training set was 0.767 and was very similar for the test set (0.759). These values are in the same range as those reported in a study inferring the regulatory network (1,934 genes; including 81 regulators) for *Halobacterium salinarum NRC-1 *[26] using 266 experimental conditions for the training model and 131 extra experiments as the test set. In this case *r *= 0.788 for the training set and *r *= 0.807 for the test set.

**Figure 5:**
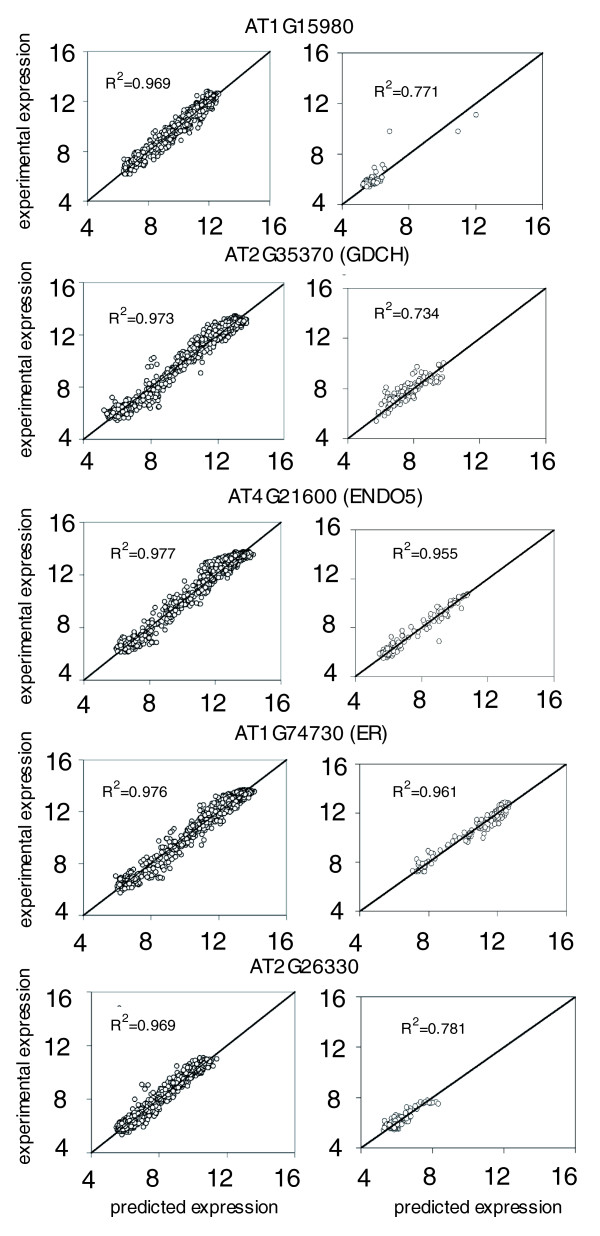
Predictive power for gene expression of the transcriptional model of *A. thaliana *inferred from the whole dataset (1,436 conditions) and the test model from 1,292 microarray experiments used as a training set. The left column shows the regression coefficient (*R*^2^) between the model and experimental profiles across the whole dataset for the five best predicted genes. The right column shows *R*^2 ^between the test model and the 144 experimental profiles used as the test set for the same five genes. In either case, correlation coefficients were highly significant.

For illustrative purposes, Figure 5 shows the expression predicted for the five best cases for the transcriptional network; each dot in the scatter plots represents a value obtained from a different hybridization experiment. The left column shows the prediction obtained using the whole dataset (1,436 experiments) as both training and tester sets, whereas the right column shows, for the same five genes, the correlation between the prediction obtained using the test model (inferred from the reduced training set of 1,292 experiments) and that obtained using the tester set (144 experiments). It is remarkable that the quality of the prediction does not change by using a reduced training set, in good agreement with the results reported for *E. coli *[25]. Similarly, Figure S8 in Additional data file 1 shows the three best and worst predicted cases for the effective gene-gene interaction model inferred from the whole dataset. In this case, the *R*^2 ^for the poorly predicted genes ranged widely, with gene *At2g02120 *(encoding a pathogenesis-related protein belonging to the defensin family) having the lowest determination coefficient observed.

### Selection of optimality in changing environments

Organisms have a high capacity for adjusting their metabolism in response to environmental changes, food availability, and developmental state [35]. On the one hand, we have detected that GO pathways (Table 4) related to response to diverse environmental (for example, defense against diverse pathogens, response to radiation, temperature, light intensity, or osmotic stress) and internal (development, secondary metabolism, porphyrin biosynthesis, and so on) stimuli consist of sets of genes with high incoming connectivity, that is, genes regulated by many different TFs. Therefore, this high degree of interconnection among different stimulus-related pathways allows the cell to rapidly adjust its homeostasis in response to changing environments. On the other hand, functional GO pathways associated with biological functions that are unaffected by external stresses (for example, glycerophospholipid and glycerophospholipid metabolic process, sulfur amino acid biosynthetic process, indole and derivative metabolic process, membrane lipid biosynthetic process, sulfured compounds biosynthetic, and Golgi vesicle transport (Table 4)), have low incoming connectivity. Notice that some GO pathways indirectly related to external stresses, such as indole derivatives (for example, camalexin, which is involved in response to the bacterium *Pseudomonas syringae*) or lipid biosynthesis pathways (playing a role in defense) do not have high connectivity scores and do not have a high number of feed-forward loops (FFLs). Furthermore, the predicted master regulators of *A. thaliana *listed in Table 2 are associated with biological functions related to transcription and regulation of cellular metabolic processes (containing 812 TFs each) or RNA metabolic processes (536 TFs) that are stimulated by environmental and developmental stresses. After all, the regulatory network of *A. thaliana *governs intracellular processes and modulates and determines the expression of the different programs encoded in the genome.

**Table 4:** Average incoming connectivity for the Gene Ontology pathways from all levels in *A. thaliana*

GO pathways*	**Number of genes**	**Number of TFs^†^**	**Number of TF/Number of genes^‡^**	**Number of FFLs^§^**
**Top five with the highest total number of TFs**				
Response to other organisms	296	2,249	7.6	9,865
Secondary metabolic process	284	1,964	6.9	3,321
Response to temperature stimulus	238	1,650	6.9	10,151
Anatomical structure morphogenesis	291	1,537	5.3	13,275
Response to radiation	250	1,524	6.1	6,233
				
**Top five with the lowest total number of TFs**				
Glycerophospholipid metabolic process	21	38	1.8	69
Sulfur amino acid biosynthetic process	24	60	2.5	13
Gametophyte development	24	62	2.6	1
Cellular morphogenesis in differentiation	25	68	2.7	78
Indole and derivative metabolic process	22	71	3.2	46
				
**Top five with the highest relative number of TFs**				
Defense response to fungus	26	355	13.7	4,353
Photosynthesis	80	1,064	13.3	2,459
Response to light intensity	26	334	12.8	2,652
Chlorophyll biosynthetic process	22	243	11.0	443
Porphyrin biosynthetic process	39	421	10.8	754
				
**Top five with the lowest relative number of TFs**				
Glycerophospolipic metabolic process	21	38	1.8	0
Membrane lipid biosynthetic process	48	111	2.3	121
Sulfur compound biosynthetic process	32	75	2.3	98
Golgi vesicle transport	44	104	2.4	47
Biogenic amine metabolic process	32	76	2.4	53

*Only GO pathways involving more than 20 genes and less than 300 from all levels were selected. ^†^Total number of TFs that regulate the genes of the GO pathway. ^‡^Relative number of TFs. ^§^Total number of FFLs involved in the GO pathway.

**Figure 6:**
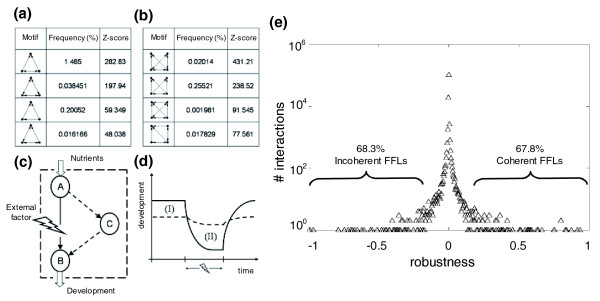
Network motifs of three **(a)** and four **(b)** genes found in the transcriptional network of *A. thaliana*. Here we plot the most statistically significant motifs (see Additional data file 2 for a complete list of motifs). **(c) **The FFL, a motif significantly overrepresented, where an external factor inhibits gene *A *thereby limiting expression of gene *B*, but this is compensated for by indirect regulation through gene *C*. **(d) **The evolution of the qualitative development of a plant with motifs (dashed line) and without motifs (solid line) under changing environments. We note that there is an evolutionary optimization, including topological units such as FFLs, that provides robustness under external factors despite decreasing system fitness (areas I and II) due to excess expression of those genes providing indirect interactions. **(e) **Distribution of normalized robustness coefficients (ρ*) computed for all interactions between TFs and genes.

Networks can be decomposed into subnetworks, which can be seen as building blocks of the former. These building blocks, generally known as motifs, are defined in terms of their frequency and are typically composed of several promoter regions of genes expressing TFs that regulate each other in a number of well known patterns (for example, bifans, forward, feed-forward, or negative feedback loops) [36]. Certain regulatory network motifs have been described as conferring robustness to perturbations in individual edges, the coherent FFL being the prototypical example of such a robustness-conferring motif [37-40]. Therefore, we sought to characterize our inferred complex network in terms of the presence and abundance of regulatory network motifs. An exhaustive list of detected three- and four-element motifs for both transcriptional regulations as well as gene-gene interactions, together with their observed frequency and whether this frequency significantly deviates from the expected value from a random network, is given in Tables S2 to S5 in Additional data file 2. Some of the overrepresented motifs are shown in Figure 6. The third most abundant motif found is the FFL (third row in Figure 6a). Indeed, the FFL motif is overrepresented among GO categories involved in stress response compared to non-stress response categories (Table 4; Fisher's exact test, *P *< 0.001).

Next, we sought to test whether the presence of FFLs indeed contributes to increase the robustness of the gene expression of the involved genes. To do so, we have computed a score, ρ*, quantifying the robustness of gene expression for all predicted TF-gene interactions involving three nodes (Figure 6c). Figure 6e shows the distribution of the robustness score computed from the inferred regulatory network. Although it may not be apparent after visual inspection of Figure 6e, the distribution is asymmetrical (skewness 1.881 ± 0.007, *P *< 0.001) and strongly leptokurtic (1,294.051 ± 0.014, *P *< 0.001), suggesting that there are more data points in the tails than close to the mean. The data points in the upper tail correspond to the more robust interactions and, if coherent FFLs are involved in such types of interactions, they may be over-represented in this tail. This is, indeed, the case. If we look at the top 1% of values, 90.7% of them correspond to a coherent FFL. By contrast, if we look at the 1% of interactions around the mean value, only 5.7% correspond to FFLs. Interestingly, 90.2% of motifs within the bottom 1% of the distribution correspond to incoherent FFLs.

## Discussion

We have discussed a reverse-engineered model of the *A. thaliana *gene regulatory network that will aid future research focused on distinguishing, for example, the molecular targets of a plant virus from the hundreds to thousands of additional gene products that may have modified levels of gene expression as a side-effect. We have used a recent methodology to infer the global topology of transcription regulation from gene expression data to produce a kinetic model able to predict the alterations in gene expression in plants subjected to different external stimuli. Moreover, we have concluded that the *A. thaliana *inferred transcriptional network presents a hierarchical scale-free architecture where biological functions cluster in modules. We have identified biological functions that are highly controlled by predicted master regulators that could change their operating points in response to dynamic external factors to produce a consistent and robust response upon different stresses at the expense of decreasing the cellular replication rate. We have successfully applied the inferred model to predict the transcriptomic response of *A. thaliana *under all experimental conditions included in the whole dataset, and also applied the test model to predict the response in a reduced test set, producing errors of 2 to 10% relative to the experimental value (averaging across all test experiments). Thus, we believe this modeling-validation approach constitutes an important step towards understanding an organism's large-scale mode of action to cope with a generally changing environment. The network model suggests that *A. thaliana *promoters are regulated by multiple TFs (Table 1), a feature that has been shown to be characteristic of eukaryotic gene regulation [2].

We have discussed a first gene regulatory model based on a transcriptional layer and a second model that enhances this by including gene-gene interactions that provide an even more accurate prediction of gene expression. Future work will consider just the interactions between tissue-specific genes. We have also quantified the presence of network motifs and found that FFLs are overwhelmingly common, thus supporting the above notion that robustness against perturbation has been a major driving force during the evolution of plant lineages. Furthermore, we have confirmed that coherent FFLs are overwhelmingly over-represented among interactions that are robust against the knockout of regulatory TFs (Figure 6e), while incoherent FFLs are among the most sensitive interactions. Figure 6c illustrates a possible mechanism by which FFLs would confer robustness. Imagine that the B product is relevant for cell survival. On one hand, regulatory flow through C is costly because it implies producing a redundant element; on the other hand, if perturbations disrupt the direct edge between A and B, the existence of C still allows the cell to obtain the precious B without incurring a major penalty (Figure 6d). Whether a given regulatory network may be selected to contain this sort of regulatory element depends on the balance between the fitness costs and benefits associated with redundancy [41,42]. The fact that *A. thaliana *network topology seems to be rich in these transcriptional regulatory elements suggests that it has been evolutionary optimized to allow rapid responses to changes in external conditions while maintaining cellular homeostasis, and hence maximizing fitness.

The reconstruction of genome-scale regulatory models constitutes a major step towards understanding cellular behavior, but it is also useful in Synthetic Biology, where predictive models can be applied to engineer synthetic systems for biotechnological applications. InferGene [25] provides a means to predict changes in biological processes when perturbing a cell in order to identify the effects of drugs, viral infection and herbicides on plant interactomes. It may also facilitate optimization of cellular processes for biotechnology applications that utilize the complex regulatory properties of genetic networks.

## Conclusions

In this study, we have shown that the *A. thaliana *regulatory network is scale-free and clustered, both characteristic properties of hierarchical networks. We also used our model to analyze the robustness of expression levels conferred by network motifs such as the coherent FFL. Hence, the meta-analysis presented here has allowed us to identify regulatory and robust genetic structures. These results suggest that *A. thaliana *has evolved a high connectivity in terms of transcriptional regulations among cellular functions involved in responses and adaptation to changing environments, while gene networks constitutively expressed or less related to stress responses are characterized by a lower connectivity. We successfully applied our quantitative network model to predict the full transcriptome of the plant for a set of microarray experiments, and the quality of the predictions was evaluated by several methods.

## Materials and methods

### Mathematical model

Gene regulations were described by a linear model based on differential equations for the dynamics of each mRNA. Data were normalized and are represented in log_2 _scale. Thus, the mRNA dynamics from the *i*^th ^gene, *y*_*i*_, is given by:



where *α *_*i *_is its constitutive transcription rate, *β *_*ij *_the regulatory effect that gene *j *has on gene *i *and *δ *_*i *_the degradation coefficient. If *j *has no effect on the expression of *i*, then *β *_*ij *_= 0. No cooperation between genes for regulation has been assumed. Time was conveniently scaled such that *δ *_*i *_= 1 and the model is assumed in steady-state (*y*_*i *_= *α *_*i *_+ Σ_*j *_*β*_*ij *_*y*_*j*_), since fitting the appropriate mRNA degradation constant would require time series data [43].

### Microarray data

Steady-state mRNA expression profiles derived from transcriptional perturbations collected from the TAIR website [44] were used in this study. We found 1,187 TFs by looking for the motif 'transcription factor' in the functionally annotated *A. thaliana *genome from TAIR (version 7). The dataset contains pre-processed expression data from 1,436 hybridization experiments using the 22,810 probe sets spotted on Affymetrix's GeneChip *Arabidopsis *ATH1 Genome Array [45]. For this study, we consider 22,094 genes. The arrays were obtained from NASCArrays [46] and AtGenExpress [47]. Data were normalized using the robust multi-array average method [27].

### Inference procedure

The inference procedure consisted of two nested steps. In the first step, the global network connectivity was inferred using the InferGene algorithm [25]. This method uses mutual information with a local significance (*z*-score computation) to obtain the genome regulations [15]. Hence, the potential interaction between a regulator and a gene is *z*-scored, constituting an estimator of the likelihood of mutual information. This approach allows some false correlations and indirect influences to be eliminated [15]. Subsequently, we selected a *z*-score threshold for a cutoff. In a second step, multiple regressions were obtained to estimate the kinetic parameters of a regulatory model based on ordinary differential equations. Multilayer models were constructed to account for different types of regulations between genes and TFs. We have constructed two different models, one for transcription regulations and another to account for effective (transcription and non-transcription) regulations. In the case of non-transcriptional interactions, Lasso's method was used to avoid over-fitting [48] and the effective interactions between genes giving the non-transcriptional layer were unveiled. To this end, we applied a simple and efficient algorithm based on the Gauss-Seidel method [49] that reduces the number of regulators that exceeded the *z*-score threshold for a given gene. Note that the Lasso method enriches in TFs among the predictors of the target for 33.21% of the non-constitutive genes of *A. thaliana *(that is, the ratio between the number of TFs selected and the total number of predictors of a given gene above a threshold defined as 1,187/22,094 = 0.0537). Finally, one systems biology markup language (SBML) [50] file containing the transcriptional model and a plain text file containing the effective model were constructed and are available as supplementary files in Additional data file 3. These files can be viewed using the Cytoscape viewer for further analysis. Notice that the transcriptional model was embedded within the effective one. Networks are constructed by placing genes as nodes and regulations as edges. For the transcriptional model, edges only go from TFs to genes (including those encoding other TFs). For the non-transcriptional model, edges connect two genes, the regulator and the target and, thus, the resulting network is directional.

### Model validation

The performance of the inferred model topology was evaluated using a reference network including genes with known transcriptional regulation. For this, the AtRegNet platform [51] linking *cis*-regulatory elements and TFs into regulatory networks was used. Only those interactions among genes included in that reference set were evaluated. The fraction of interactions that were correctly predicted by the model (precision, *P*) and the fraction of all known interactions that were discovered by the model (sensitivity, *S*) were used to compute a performance statistic defined as *F *= 2*PS*/(*P *+ *S*) [16]. We have to note that the number of transcriptional regulations experimentally confirmed and compiled in AtRegNet is quite limited, containing only 448 reported interactions between TFs and genes. Therefore, it is difficult to obtain an accurate value for the performance of the model.

To validate the predictive power of the methodology, we constructed two transcription models. The first was obtained by using the 1,436 microarrays for training. For the second model (the test model), 1,292 of these 1,436 microarrays were used as a training set (90%) and 144 randomly chosen ones (10%) were retained for validation studies.

### Motif detection and analysis

The FANDOM program [52] was used to detect motifs of three and four genes in the predicted *A. thaliana *regulatory model. Statistically significant motifs have *z*-scores > 2.

The robustness of gene expression to perturbations in the underlying motifs was evaluated for each interaction as follows. In the scheme illustrated in Figure 6c, TF *A *operates on gene *B *but also may act upon a second TF *C *that, itself, may also interact with the promoter region of *B*, activating its expression. For such a system, we define the robustness score to quantify the impact that removing TF *A *has on the expression of gene *B*:



where  represents the measured expression of gene *B *when gene *A *is present and  after it has been removed. The difference in gene expression is normalized by the expression level of the TF *A*, *y*_*A*_, and the strength of its regulation, *β*_*AB*_, on the expression of *B*. If *A *is removed (*y*_*A *_→ 0) and no alternative pathway exists, then *ρ*_*AB *_→ 1. However, if *C *exists, as is the case for FFLs, then *ρ*_*AB *_≠ 1, with its sign being determined by  and the sign of *β*_*AB*_. This score is unbounded; thus, for convenience we further normalize it as:



which is now contained in the interval [-1, 1]. Values of  close to 1 would correspond to maximally robust motifs, whereas values close to zero correspond to motifs not contributing to the robustness of the network. Values close to -1 correspond to incoherent motifs, that is, gene circuits implementing antagonistic regulations [34].

## Abbreviations

*F*: absolute efficiency; FFL: feed-forward loop; GO: Gene Ontology; *P*: precision; *S*: sensitivity; TAIR: The *Arabidopsis *Information Resource; TF: transcription factor.

## Authors' contributions

SFE conceived the study. JC, GR and AJ performed all the computations. All authors analyzed the data and contributed to writing the manuscript.

## Additional data files

The following additional data are available with the online version of this paper: Figures S1 to S8 (Additional data file 1); Tables S1 to S5 (Additional data file 2); the effective and transcriptional models (Additional data file 3).

## Supplementary Material

Additional data file 1 Figure S1: *z*-score distribution from the mutual information calculation between all gene-TF pairs. Figure S2: number of regulations in the model depending on the cutoff threshold selection. Figure S3: efficiency (precision, sensitivity and *F*-score) of the transcriptional model with respect to the reference set. The vertical dashed line indicates the optimum value for the *z*-score threshold (= 5) according to the *F *value. Figure S4: gene distribution in the pathways (clusters) found in the transcriptional network. Figure S5: stress distribution of the transcriptional network. Figure S6: absolute and relative gene expression errors versus the regression coefficient between the experimental and predicted gene expressions for all conditions from the training set. Figure S7: regression coefficient between the experimental and predicted gene expressions for all conditions versus the number of TFs regulating that gene. Figure S8: predictive power for gene expression of the effective model (including the transcriptional and non-transcriptional layers). We show the regression coefficient (*R*^2^) between the model and experimental profiles across the 1,436 conditions for the best (top) and worst (bottom) predicted genes. Click here for file

Additional data file 2 Table S1: fit of the distributions of outgoing and incoming connectivities for the transcriptional and non-transcriptional models to different statistical distributions. Table S2: three-gene motifs for the transcriptional model showing abundance and statistical significance. Table S3: four-gene motifs for the transcriptional model showing abundance and statistical significance. Table S4: three-gene motifs for the non-transcriptional model showing abundance and statistical significance. Table S5: four-gene motifs for the non-transcriptional model showing abundance and statistical significance. Click here for file

Additional data file 3 The effective (text file) and transcriptional models (SBML file). Click here for file
