# Supplementary material for: Reverse-engineering the Arabidopsis thaliana transcriptional network under changing environmental conditions
Source: Genome Biol. 2009 Sep 15;10(9):R96. doi: 10.1186/gb-2009-10-9-r96 (PMC2768985; doi:10.1186/gb-2009-10-9-r96)
Supplement: Additional data file 1 — Figure S1: z-score distribution from the mutual information calculation between all gene-TF pairs. Figure S2: number of regulations in the model depending on the cutoff threshold selection. Figure S3: efficiency (precision, sensitivity and F-score) of the transcriptional model with respect to the reference set. The vertical dashed line indicates the optimum value for the z-score threshold (= 5) according to the F value. Figure S4: gene distribution in the pathways (clusters) found in the transcriptional network. Figure S5: stress distribution of the transcriptional network. Figure S6: absolute and relative gene expression errors versus the regression coefficient between the experimental and predicted gene expressions for all conditions from the training set. Figure S7: regression coefficient between the experimental and predicted gene expressions for all conditions versus the number of TFs regulating that gene. Figure S8: predictive power for gene expression of the effective model (including the transcriptional and non-transcriptional layers). We show the regression coefficient (R2) between the model and experimental profiles across the 1,436 conditions for the best (top) and worst (bottom) predicted genes. [file gb-2009-10-9-r96-S1.PDF]

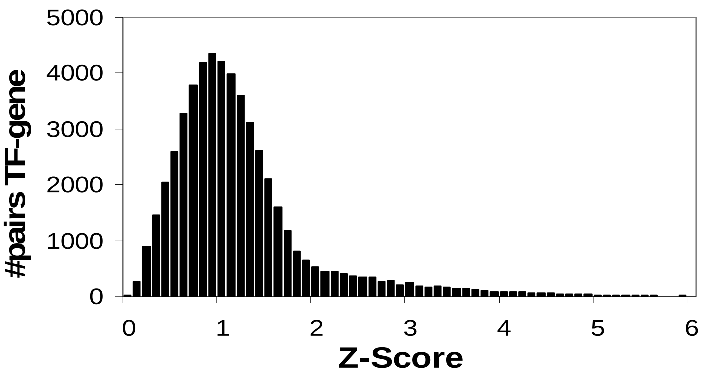

Supplementary Figure 1. *z*-score distribution from the mutual information calculation between all pairs gene-transcription factor.

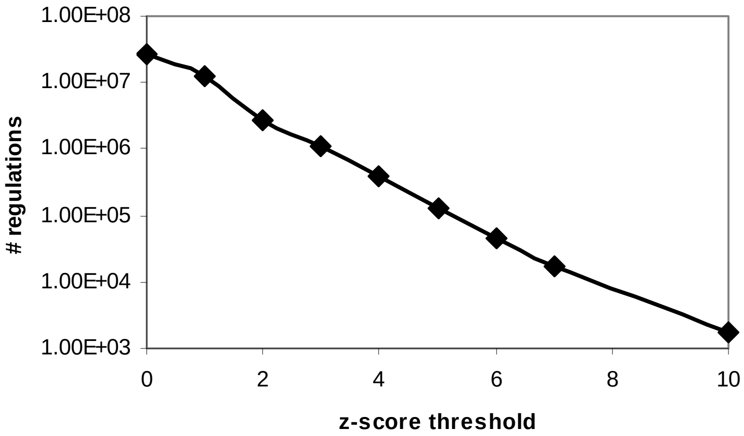

Supplementary Figure 2. Number of regulations of model depending on the cut-off threshold selection.

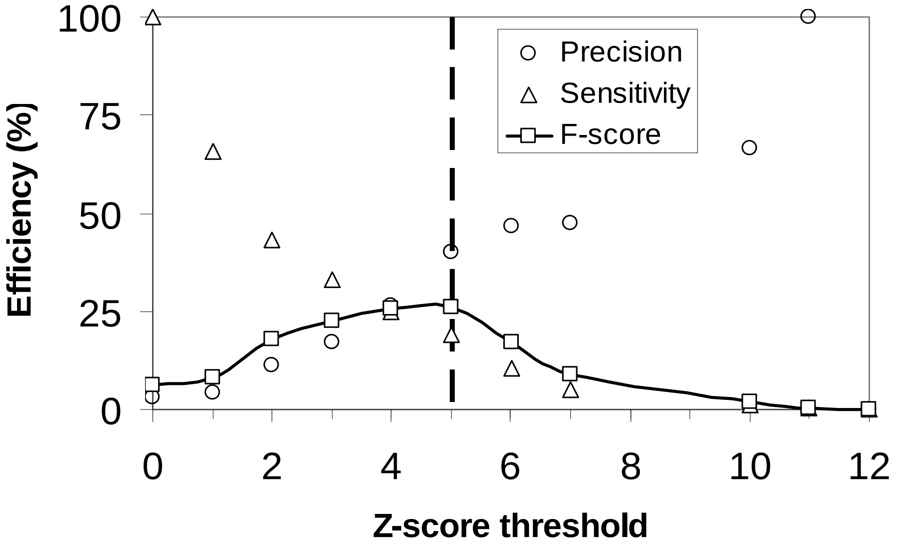

Supplementary Figure 3. Efficiency (precision, sensitivity and absolute efficiency or *F*-score) of the transcriptional model with respect to reference set. Vertical dashed line indicates the optimum value for the *z*-score threshold (= 5) according to the *F* value.

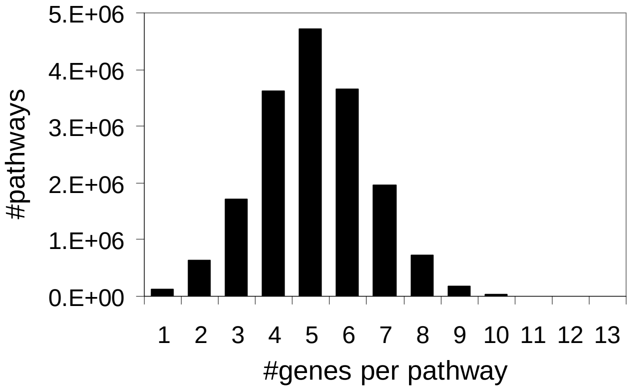

Supplementary Figure 4. Gene distribution in the pathways (clusters) found in the transcriptional network.

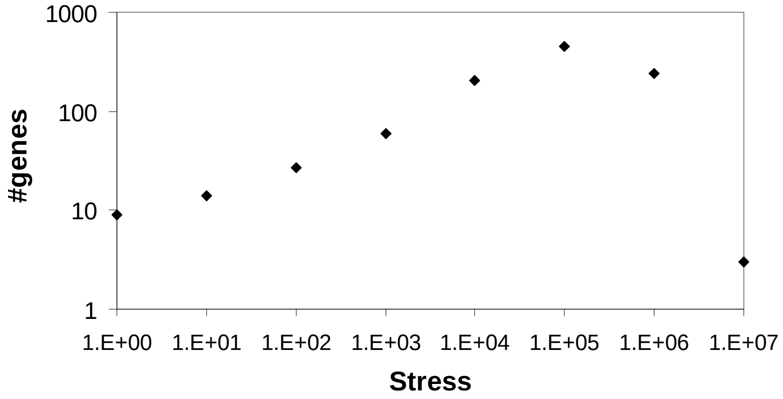

Supplementary Figure 5. Stress distribution of the transcriptional network.

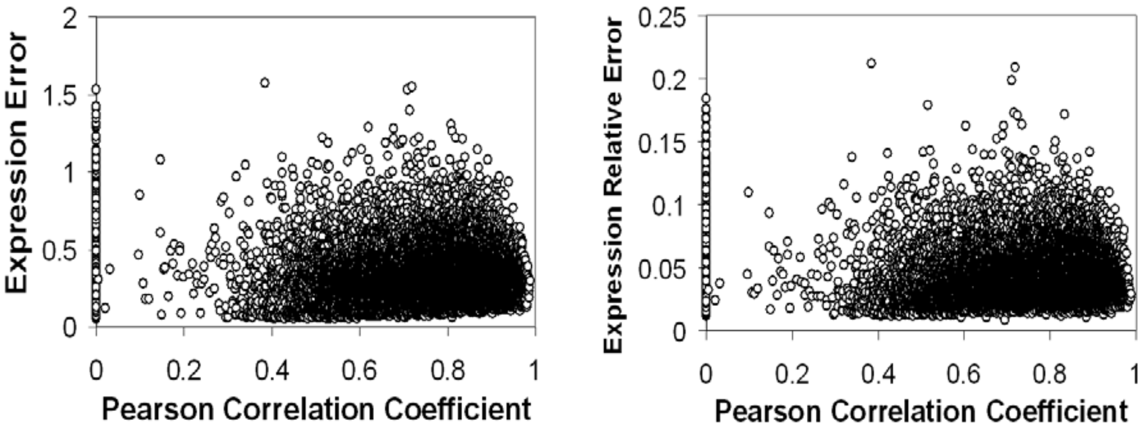

Supplementary Figure 6. Absolute and relative gene expression errors versus the correlation coefficient between the experimental and predicted gene expressions for all conditions from the training set.

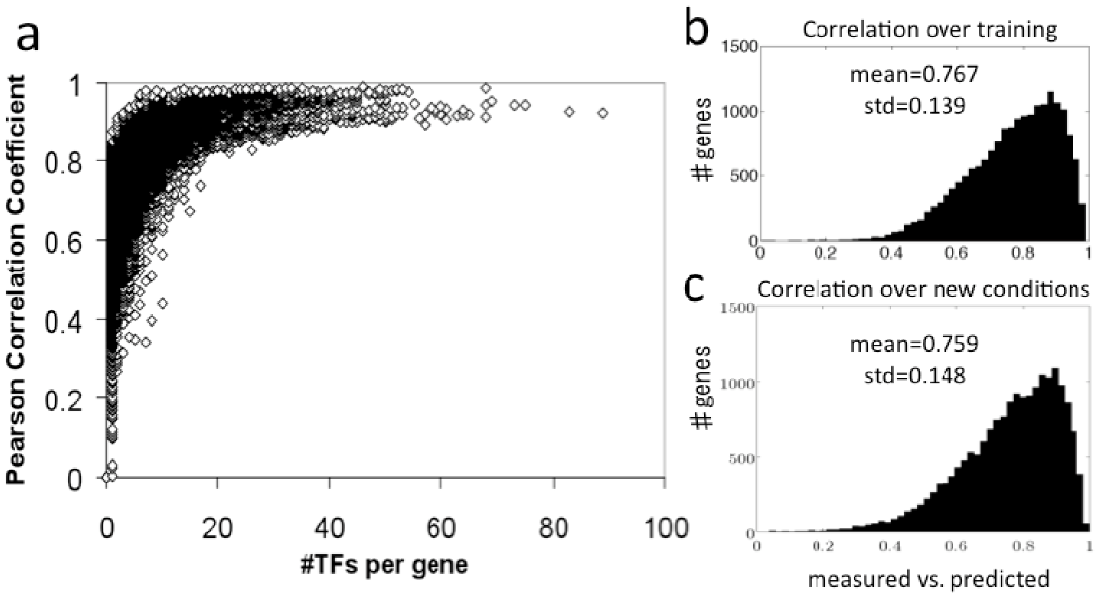

Supplementary Figure 7. (a) Correlation coefficient between the experimental and predicted gene expressions for all conditions versus the number of transcription factors regulating that gene. (b, c) Prediction in different environments using the transcriptional regulatory network. Histogram of Pearson correlations of measured and predicted gene expressions over 1292 experiments in the training set (b) and 144 conditions in the test set (b) are shown.

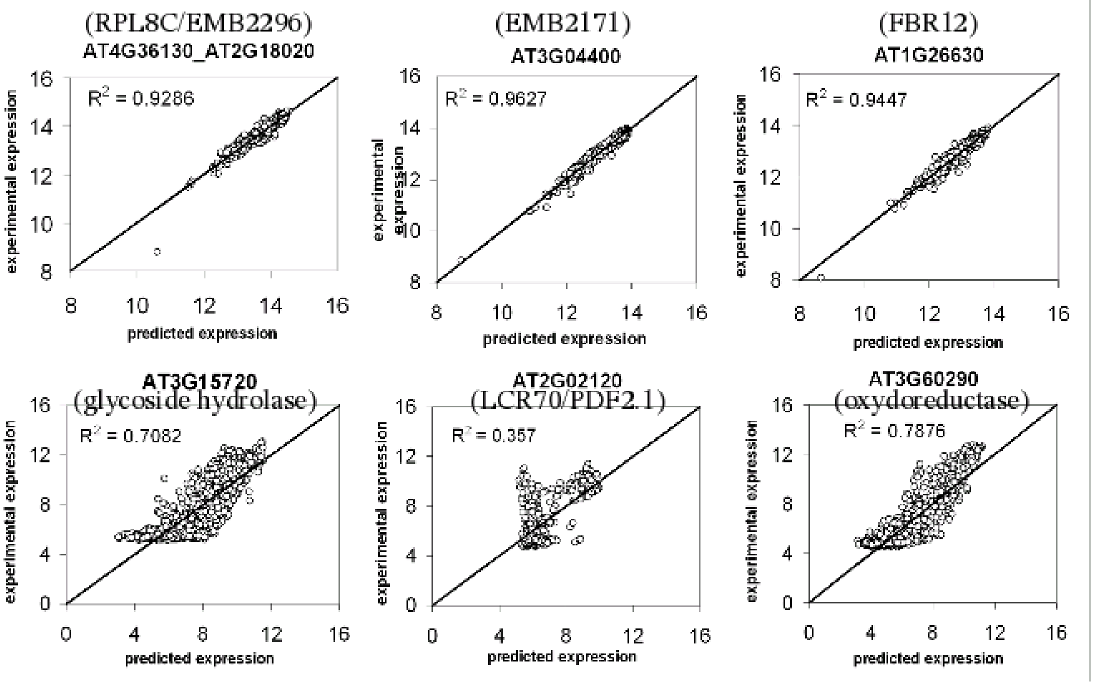

Supplementary Figure 8. Predictive power on gene expression of the effective model (including the transcriptional and non-transcriptional layers). We show the regression coefficient (R^2) between the model inferred using the whole data (1436 experiments) both as training and as tester sets for the best (top) and worst (bottom) predicted genes.
