# Supplementary material for: Reverse-engineering the Arabidopsis thaliana transcriptional network under changing environmental conditions
Source: Genome Biol. 2009 Sep 15;10(9):R96. doi: 10.1186/gb-2009-10-9-r96 (PMC2768985; doi:10.1186/gb-2009-10-9-r96)
Supplement: Additional data file 2 — Table S1: fit of the distributions of outgoing and incoming connectivities for the transcriptional and non-transcriptional models to different statistical distributions. Table S2: three-gene motifs for the transcriptional model showing abundance and statistical significance. Table S3: four-gene motifs for the transcriptional model showing abundance and statistical significance. Table S4: three-gene motifs for the non-transcriptional model showing abundance and statistical significance. Table S5: four-gene motifs for the non-transcriptional model showing abundance and statistical significance. [file gb-2009-10-9-r96-S2.PDF]

| Data set | Distribution | Parameter values±SE | $R^2$ | Log-likelihood | Akaike's weight |
|---|---|---|---|---|---|
| TFs outcoming connectivity (Fig 2a) | Exponential | scale = 0.010±0.000 | 0.996 | 4609.024 | 0 |
| | Normal | mean = 78.098±0.525<br>SD = 77.648±0.902 | 0.948 | 3178.376 | 0 |
| | Half-Normal | mean = –13.197±0.427<br>SD = 127.440±0.795 | 0.981 | 3776.068 | 0 |
| | Gamma | shape = 0.859±0.002<br>scale = 0.008±0.000 | 0.999 | 5505.990 | $9.779\ 10^{-92}$ |
| | Beta | shape1 = 0.819±0.002<br>shape2 = 9.536±0.041 | 0.998 | 5186.236 | $1.328\ 10^{-230}$ |
| | Laplace | location = 75.629±0.525<br>scale = 72.934±0.998 | 0.949 | 3186.7639 | 0 |
| | Log-Normal | scale = 61.179±0.151<br>shape = 1.283±0.004 | 0.995 | 4579.751 | 0 |
| | Pareto | scale = 14.768±0.199<br>shape = 0.563±0.006 | 0.983 | 3076.681 | 0 |
| | Weibull | cut-off = 99.093±0.093<br>shape = 0.902±0.001 | 0.999 | 5715.547 | 1 |
| | Power law | scale = 393.801±5.832<br>shape = 0.409±0.005 | 0.906 | 2827.129 | 0 |
| | Lévy | scale = 0.035±0.000<br>shape = –1.591±0.005 | 0.906 | 2827.129 | 0 |
| TFs incoming connectivity (Fig 2b) | Exponential | scale = 0.200±0.000 | 0.965 | 64694.892 | 0 |
| | Normal | mean = 4.433±0.012<br>SD = 4.032±0.007 | 0.952 | 61045.884 | 0 |
| | Half-Normal | mean = –1.226±0.005<br>SD = 7.370±0.010 | 0.983 | 72454.697 | 1 |
| | Gamma | shape = 0.119±0.000<br>scale = 0.682±0.002 | 0.982 | 72229.749 | $2.026\ 10^{-98}$ |
| | Beta | shape1 = 0.654±0.002<br>shape2 = 9.782±0.037 | 0.982 | 72119.989 | $4.350\ 10^{-146}$ |
| | Laplace | location = 3.895±0.007<br>scale = 4.180±0.013 | 0.954 | 61482.200 | 0 |
| | Log-Normal | scale = 3.045±0.004<br>shape = 1.414±0.003 | 0.977 | 69238.636 | 0 |
| | Pareto | scale = 1.152±0.007<br>shape = 0.734±0.004 | 0.863 | 49500.360 | 0 |
| | Weibull | cut-off = 5.192±0.006<br>shape = 0.784±0.001 | 0.982 | 72153.399 | $1.406\ 10^{-131}$ |
| | Power law | scale = 22.600±0.073<br>shape = 0.359±0.001 | 0.941 | 58894.261 | 0 |
| | Lévy | scale = 0.117±0.000<br>shape = –1.641±0.001 | 0.941 | 58894.261 | 0 |
| Genes outcoming connectivity (Fig 2e) | Exponential | scale = 0.049±0.000 | 0.976 | 68520.194 | 0 |
| | Normal | mean = 16.347±0.031<br>SD = 17.647±0.054 | 0.936 | 57854.525 | 0 |
| | Half-Normal | mean = –4.345±0.028<br>SD = 29.025±0.054 | 0.971 | 66739.912 | 0 |
| | Gamma | shape = 0.669±0.000<br>scale = 0.028±0.000 | 0.998 | 93736.373 | 0 |
| | Beta | shape1 = 0.662±0.001 | 0.997 | 93035.694 | 0 |

| | | | | | |
|---|---|---|---|---|---|
| | | shape2 = 34.647±0.049 | | | |
| | Laplace | location = 15.758±0.031<br>scale = 16.732±0.060 | 0.939 | 58330.312 | 0 |
| | Log-Normal | scale = 12.234±0.010<br>shape = 1.492±0.001 | 0.993 | 81918.410 | 0 |
| | Pareto | scale = 1.992±0.008<br>shape = 0.441±0.001 | 0.929 | 56626.553 | 0 |
| | Weibull | cut-off = 21.341±0.009<br>shape = 0.765±0.000 | 0.998 | 96413.862 | 1 |
| | Power law | scale = 3.914±0.009<br>shape = 1.316±0.001 | 0.900 | 52890.428 | 0 |
| | Lévy | scale = 0.069±0.000<br>shape = –1.684±0.001 | 0.900 | 52890.428 | 0 |
| Genes incoming connectivity (Fig 2f) | Exponential | scale = 0.028±0.000 | 0.868 | 49799.864 | 0 |
| | Normal | mean = 15.768±0.008<br>SD = 28.197±0.004 | 0.998 | 97008.307 | 1 |
| | Half-Normal | mean = 8.859±0.019<br>SD = 26.928±0.029 | 0.989 | 77589.399 | 0 |
| | Gamma | shape = 3.280±0.005<br>scale = 0.108±0.000 | 0.995 | 85458.853 | 0 |
| | Beta | shape1 = 2.421±0.003<br>shape2 = 6.536±0.008 | 0.997 | 93029.476 | 0 |
| | Laplace | location = 28.026±0.008<br>scale = 14.287±0.015 | 0.994 | 83998.730 | 0 |
| | Log-Normal | scale = 26.832±0.011<br>shape = 0.564±0.001 | 0.988 | 76467.457 | 0 |
| | Pareto | scale = 14.756±0.022<br>shape = 1.328±0.004 | 0.931 | 56918.340 | 0 |
| | Weibull | cut-off = 33.211±0.006<br>shape = 1.985±0.001 | 0.998 | 95498.035 | 0 |
| | Power law | scale = 0.018±0.000<br>shape = 0.007±0.002 | 0.957 | 62333.447 | 0 |
| | Lévy | scale = 0.018±0.000<br>shape = –1.007±0.002 | 0.957 | 62333.447 | 0 |

Supplementary Table 1. Fit of the distributions of outcoming and incoming connectivities for the transcriptional and non-transcriptional models to different statistical distributions. The Exponential, Normal, Half-Normal, Gamma, and Beta belong to the exponential-like family. The Laplace, Log-Normal, Pareto, Weibull, Power law and Lévy to the heavy-tail or stretched exponential-like family.

**Network motifs**

**MOTIFS OF 3 GENES FOR TRANSCRIPTIONAL NETWORK**

| Adj | Frequency [Original] | Mean-Freq [Random] | Standard-Dev [Random] | Z-Score |
|---|---|---|---|---|
| 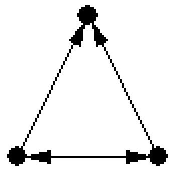 | 1.485% | 0.084724% | 4.9509e-005 | 282.83 |
| 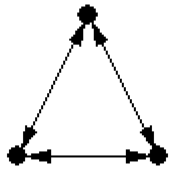 | 0.038451% | 0.00012282% | 1.9363e-006 | 197.94 |
| 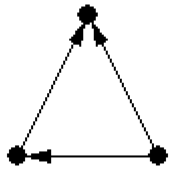 | 0.20052% | 0.034328% | 2.8002e-005 | 59.349 |
| 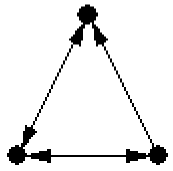 | 0.016166% | 0.0016292% | 3.0261e-006 | 48.038 |
| 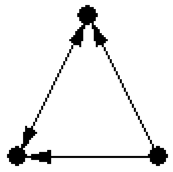 | 0.00235% | 0.00016271% | 8.0979e-007 | 27.01 |
| 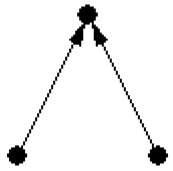 | 2.6139% | 4.1352% | 0.0012183 | -12.487 |

| | | | | |
|---|---|---|---|---|
| 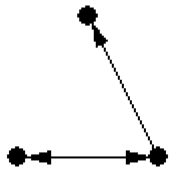 | 5.2916% | 7.3553% | 0.0037624 | -5.4852 |
| 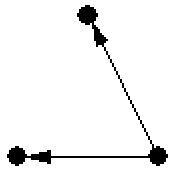 | 89.196% | 86.975% | 0.0052734 | 4.2116 |
| 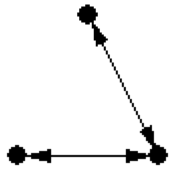 | 0.16349% | 0.23966% | 0.00020959 | -3.6346 |
| 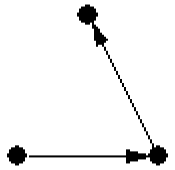 | 0.93448% | 1.1051% | 0.00064718 | -2.6361 |
| 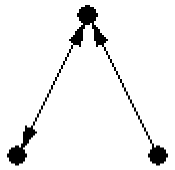 | 0.057737% | 0.068493% | 6.2336e-005 | -1.7256 |
| 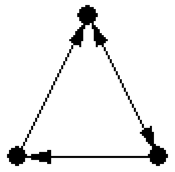 | 0.00032414% | 0.00020487% | 1.0626e-006 | 1.1224 |

Supplementary Table 2: Motifs of 3 genes for the transcriptional model showing the abundance and the statistical significance.

## MOTIFS OF 4 GENES FOR TRANSCRIPTIONAL NETWORK

| Adj | Frequency [Original] | Mean-Freq [Random] | Standard-Dev [Random] | Z-Score |
|---|---|---|---|---|
| 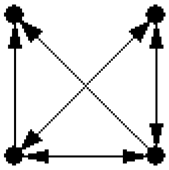 | 0.02014% | 8.1263e-006% | 4.6687e-007 | 431.21 |
| 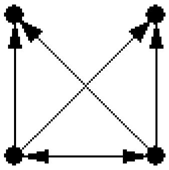 | 0.25521% | 0.0014801% | 1.0638e-005 | 238.52 |
| 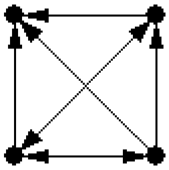 | 0.001981% | 2.1615e-006% | 2.1615e-007 | 91.545 |
| 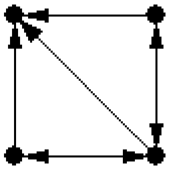 | 0.017829% | 0.00020269% | 2.2725e-006 | 77.561 |
| 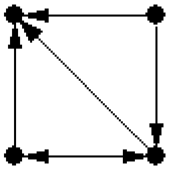 | 0.0059429% | 5.4126e-005% | 1.2212e-006 | 48.222 |
| 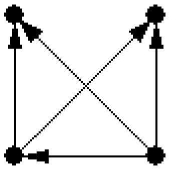 | 0.024432% | 0.00062174% | 5.6444e-006 | 42.183 |
| 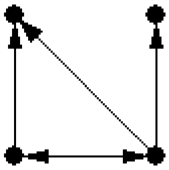 | 1.8902% | 0.22282% | 0.00061292 | 27.203 |
| 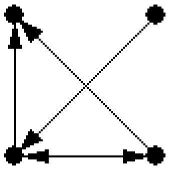 | 0.029054% | 0.0017006% | 1.2115e-005 | 22.577 |

| | | | | |
|---|---|---|---|---|
| 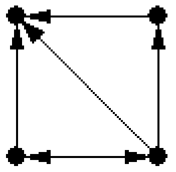 | 0.0033016% | 6.644e-005% | 1.5069e-006 | 21.469 |
| 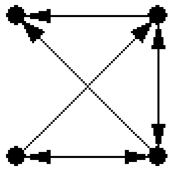 | 0.0042921% | 3.8553e-005% | 2.0259e-006 | 20.996 |
| 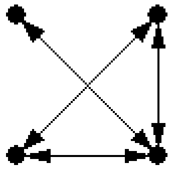 | 0.0036318% | 4.7423e-005% | 2.1538e-006 | 16.642 |
| 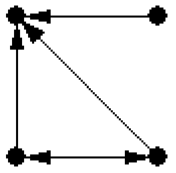 | 0.054146% | 0.0083173% | 2.8619e-005 | 16.013 |
| 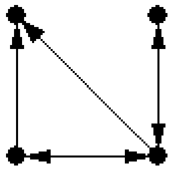 | 0.088153% | 0.012923% | 4.8339e-005 | 15.563 |
| 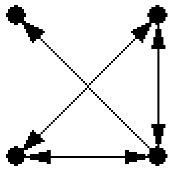 | 0.058768% | 0.00097664% | 4.1714e-005 | 13.854 |
| 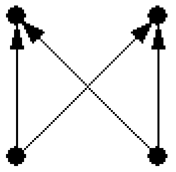 | 0.2813% | 0.086394% | 0.00018975 | 10.271 |
| 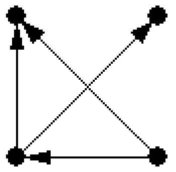 | 0.22286% | 0.043975% | 0.00020062 | 8.9165 |

| | | | | |
|---|---|---|---|---|
| 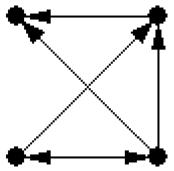 | 0.00033016% | 5.284e-006% | 3.7545e-007 | 8.6529 |
| 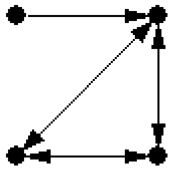 | 0.00099048% | 1.6984e-005% | 1.2001e-006 | 8.1119 |
| 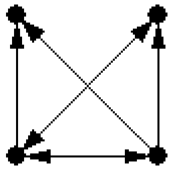 | 0.00099048% | 4.2739e-005% | 1.415e-006 | 6.6977 |
| 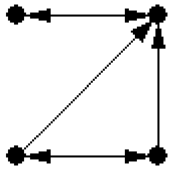 | 0.00033016% | 9.0268e-006% | 5.1606e-007 | 6.2227 |
| 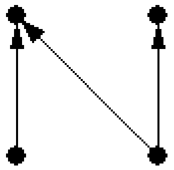 | 5.164% | 10.224% | 0.009983 | -5.0682 |
| 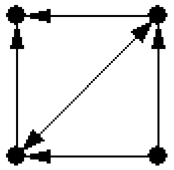 | 0.00099048% | 2.8265e-005% | 1.9398e-006 | 4.9603 |
| 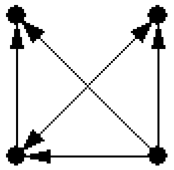 | 0.00033016% | 1.7072e-005% | 6.8505e-007 | 4.5703 |
| 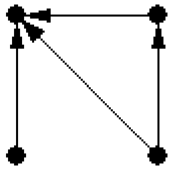 | 0.010565% | 0.0035627% | 1.8294e-005 | 3.8276 |

| | | | | |
|---|---|---|---|---|
| 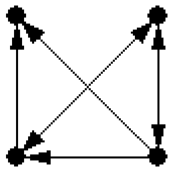 | 0.00033016% | 2.1459e-005% | 8.5062e-007 | 3.6291 |
| 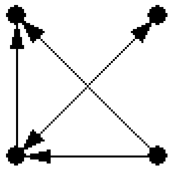 | 0.0075937% | 0.0024795% | 1.4093e-005 | 3.6289 |
| 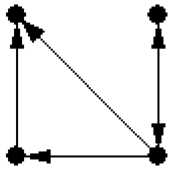 | 0.0066032% | 0.0021507% | 1.26e-005 | 3.5337 |
| 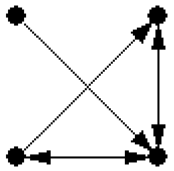 | 0.00033016% | 2.7289e-005% | 8.7783e-007 | 3.4502 |
| 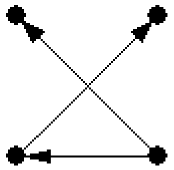 | 0.44638% | 1.4051% | 0.0028218 | -3.3976 |
| 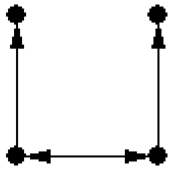 | 1.4596% | 3.3845% | 0.0057297 | -3.3595 |
| 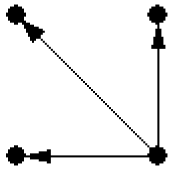 | 82.769% | 74.103% | 0.027329 | 3.1711 |
| 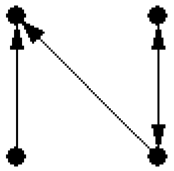 | 0.19512% | 0.45008% | 0.00083837 | -3.0411 |

| | | | | |
|---|---|---|---|---|
| 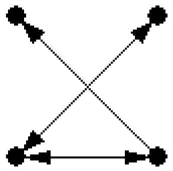 | 0.17829% | 0.44786% | 0.0011186 | -2.41 |
| 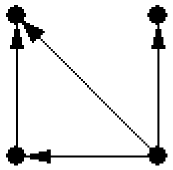 | 0.11688% | 0.063209% | 0.00024644 | 2.1777 |
| 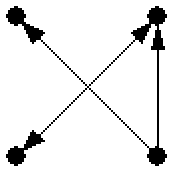 | 0.023771% | 0.08586% | 0.00028882 | -2.1497 |
| 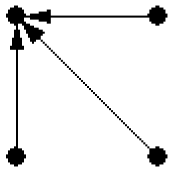 | 0.10334% | 0.18737% | 0.00039894 | -2.1062 |
| 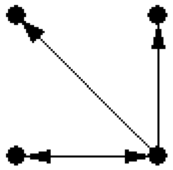 | 4.6751% | 7.4176% | 0.013263 | -2.0679 |
| 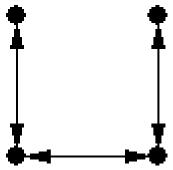 | 0.0042921% | 0.015549% | 5.5229e-005 | -2.0382 |
| 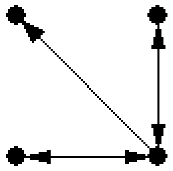 | 0.18522% | 0.40888% | 0.0011301 | -1.9791 |
| 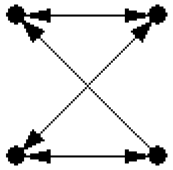 | 0.00033016% | 6.5489e-005% | 1.3472e-006 | 1.9646 |

| | | | | |
|---|---|---|---|---|
| 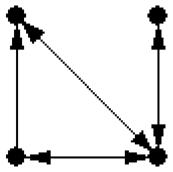 | 0.00066032% | 0.00015947% | 2.7708e-006 | 1.8076 |
| 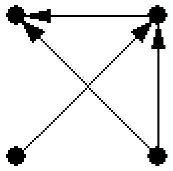 | 0.00099048% | 0.00036015% | 3.7876e-006 | 1.6642 |
| 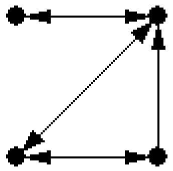 | 0.00066032% | 0.00015212% | 3.2075e-006 | 1.5844 |
| 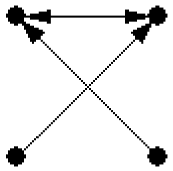 | 0.00066032% | 0.00021913% | 2.7948e-006 | 1.5786 |
| 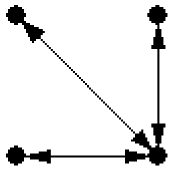 | 0.0039619% | 0.010447% | 4.3379e-005 | -1.4949 |
| 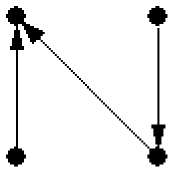 | 0.038959% | 0.063091% | 0.00017365 | -1.3897 |
| 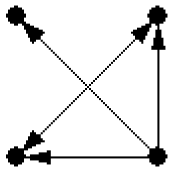 | 0.00066032% | 0.00016903% | 3.8341e-006 | 1.2814 |
| 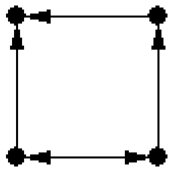 | 0.00033016% | 0.0010826% | 7.0796e-006 | -1.0628 |

| | | | | |
|---|---|---|---|---|
| 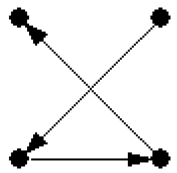 | 0.0036318% | 0.0085744% | 5.7912e-005 | -0.85346 |
| 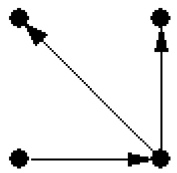 | 1.3685% | 1.0666% | 0.0037914 | 0.79618 |
| 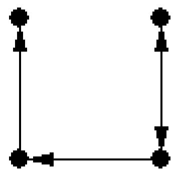 | 0.043251% | 0.057708% | 0.00021368 | -0.67656 |
| 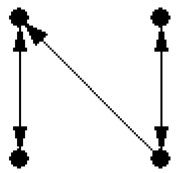 | 0.0023111% | 0.0035489% | 1.9246e-005 | -0.64312 |
| 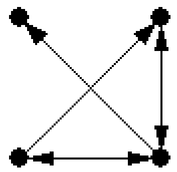 | 0.0036318% | 0.0018051% | 2.9876e-005 | 0.61141 |
| 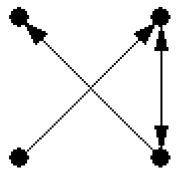 | 0.046883% | 0.058976% | 0.00023019 | -0.52535 |
| 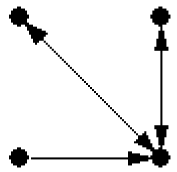 | 0.0052826% | 0.0042652% | 2.3963e-005 | 0.42454 |
| 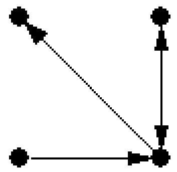 | 0.12777% | 0.10973% | 0.00045192 | 0.39918 |

| | | | | |
|---|---|---|---|---|
| 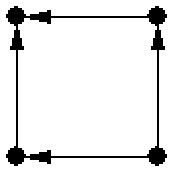 | 0.00033016% | 0.0011681% | 2.3118e-005 | -0.36247 |
| 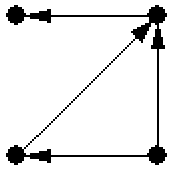 | 0.00033016% | 0.00010688% | 6.1987e-006 | 0.36021 |
| 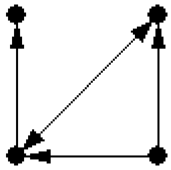 | 0.0016508% | 0.00054644% | 3.2095e-005 | 0.34409 |
| 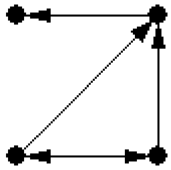 | 0.00033016% | 0.00015403% | 5.7387e-006 | 0.30692 |
| 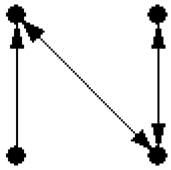 | 0.0033016% | 0.0038746% | 2.175e-005 | -0.26346 |
| 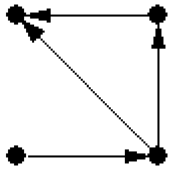 | 0.00033016% | 0.00025388% | 3.5418e-006 | 0.21536 |
| 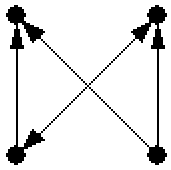 | 0.0016508% | 0.0014868% | 8.5106e-006 | 0.19268 |
| 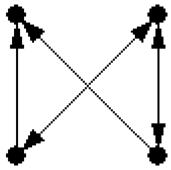 | 0.0069333% | 0.0077876% | 4.4548e-005 | -0.19177 |

| | | | | |
|---|---|---|---|---|
| 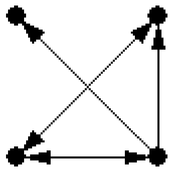 | 0.001981% | 0.0024938% | 3.5381e-005 | -0.14494 |
| 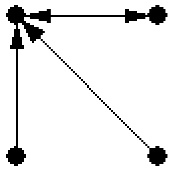 | 0.00066032% | 0.00073615% | 6.1446e-006 | -0.12342 |
| 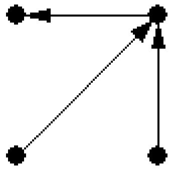 | 0.010565% | 0.0098879% | 5.9354e-005 | 0.1141 |
| 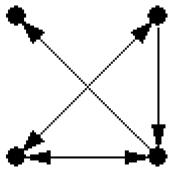 | 0.0023111% | 0.0020555% | 4.0015e-005 | 0.063875 |
| 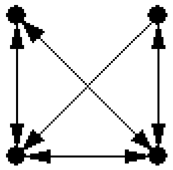 | 0.00066032% | 0% | 0 | undefined |
| 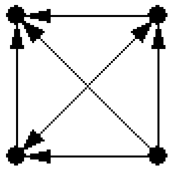 | 0.00066032% | 0% | 0 | undefined |
| 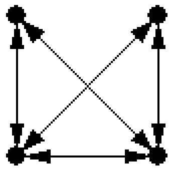 | 0.00099048% | 0% | 0 | undefined |
| 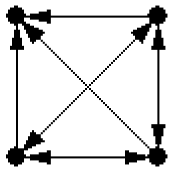 | 0.0072635% | 0% | 0 | undefined |

Supplementary Table 3: Motifs of 4 genes for the transcriptional model showing the abundance and the statistical significance.

## MOTIFS OF 3 GENES FOR NON-TRANSCRIPTIONAL NETWORK

| Adj | Frequency [Original] | Mean-Freq [Random] | Standard-Dev [Random] | Z-Score |
|---|---|---|---|---|
| 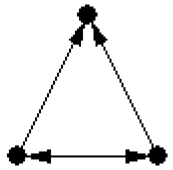 | 0.39429% | 0.0044641% | 3.4676e-006 | 1124.2 |
| 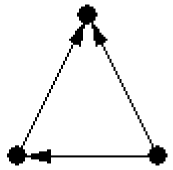 | 1.615% | 0.075068% | 1.839e-005 | 837.42 |
| 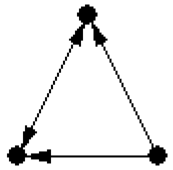 | 0.65186% | 0.022414% | 8.3882e-006 | 750.4 |
| 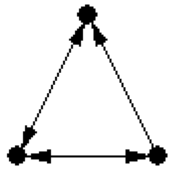 | 0.41017% | 0.008637% | 5.9211e-006 | 678.13 |
| 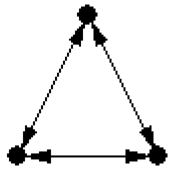 | 0.23711% | 0.00029255% | 3.7978e-006 | 623.56 |
| 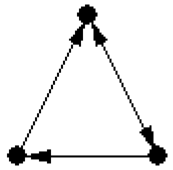 | 0.060358% | 0.0043988% | 3.3795e-006 | 165.58 |
| 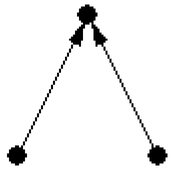 | 11.024% | 12.276% | 0.0010269 | -12.192 |
| 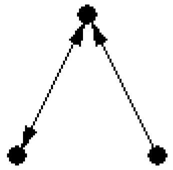 | 8.0271% | 9.1071% | 0.0011833 | -9.1274 |

| | | | | |
|---|---|---|---|---|
| 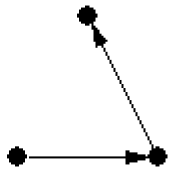 | 10.264% | 11.159% | 0.0012737 | -7.02 |
| 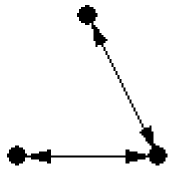 | 4.8809% | 5.5055% | 0.0012816 | -4.8739 |
| 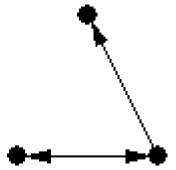 | 8.0128% | 8.5608% | 0.0014471 | -3.787 |
| 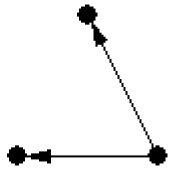 | 54.421% | 53.276% | 0.0048679 | 2.353 |
| 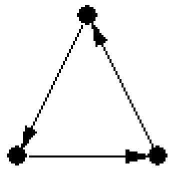 | 0.000929% | 0.0011901% | 1.8504e-006 | -1.4112 |

Supplementary Table 4: Motifs of 3 genes for the non-transcriptional model showing the abundance and the statistical significance.

## MOTIFS OF 4 GENES FOR NON-TRANSCRIPTIONAL NETWORK

| Adj | Frequency [Original] | Mean-Freq [Random] | Standard-Dev [Random] | Z-Score |
|---|---|---|---|---|
| 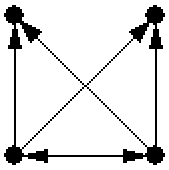 | 0.049273% | 2.3308e-005% | 7.9634e-007 | 618.45 |
| 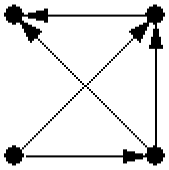 | 0.011767% | 2.7394e-006% | 2.7394e-007 | 429.44 |
| 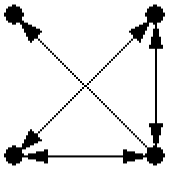 | 0.052215% | 5.5943e-005% | 1.4937e-006 | 349.18 |
| 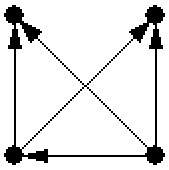 | 0.064717% | 0.00029333% | 3.0131e-006 | 213.81 |
| 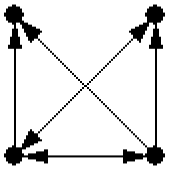 | 0.0058833% | 2.9742e-006% | 2.9742e-007 | 197.71 |
| 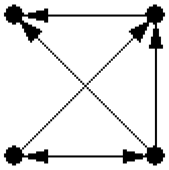 | 0.0073542% | 5.5382e-006% | 3.8969e-007 | 188.58 |
| 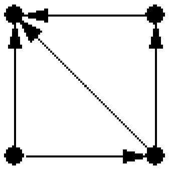 | 0.0080896% | 8.704e-006% | 4.9955e-007 | 161.76 |
| 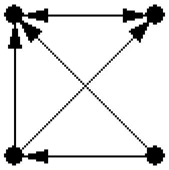 | 0.006251% | 5.9526e-006% | 4.1924e-007 | 148.96 |

| | | | | |
|---|---|---|---|---|
| 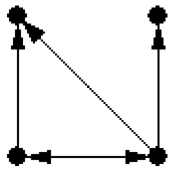 | 0.6137% | 0.0054218% | 4.2682e-005 | 142.52 |
| 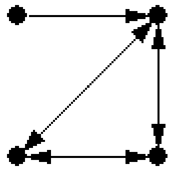 | 0.041919% | 0.00010514% | 2.987e-006 | 139.98 |
| 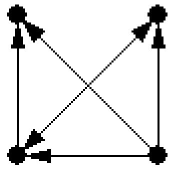 | 0.014708% | 4.3201e-005% | 1.0916e-006 | 134.35 |
| 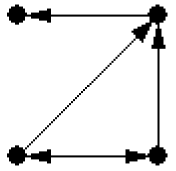 | 0.041183% | 0.00022922% | 3.095e-006 | 132.32 |
| 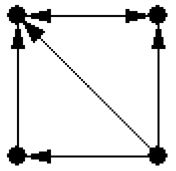 | 0.013237% | 3.3153e-005% | 1.0581e-006 | 124.8 |
| 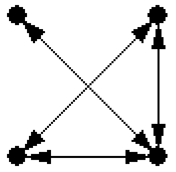 | 0.065084% | 0.00019489% | 5.2619e-006 | 123.32 |
| 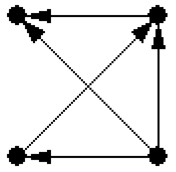 | 0.013605% | 5.125e-005% | 1.1059e-006 | 122.56 |
| 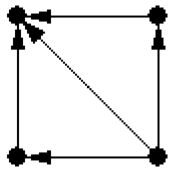 | 0.025004% | 0.00012909% | 2.1012e-006 | 118.39 |

| | | | | |
|---|---|---|---|---|
| 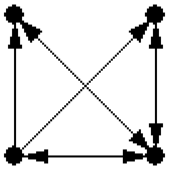 | 0.006251% | 1.3596e-005% | 5.9667e-007 | 104.54 |
| 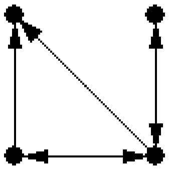 | 0.086779% | 0.0010671% | 8.5456e-006 | 100.3 |
| 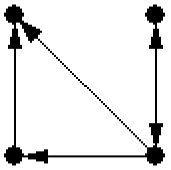 | 0.12576% | 0.0040112% | 1.4003e-005 | 86.942 |
| 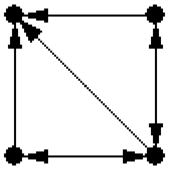 | 0.0033094% | 5.6009e-006% | 3.9592e-007 | 83.446 |
| 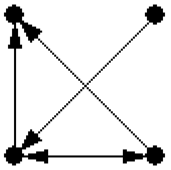 | 0.040816% | 0.00061243% | 4.8769e-006 | 82.437 |
| 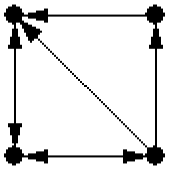 | 0.0029417% | 5.2427e-006% | 3.6907e-007 | 79.562 |
| 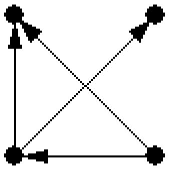 | 0.58245% | 0.029403% | 7.0408e-005 | 78.549 |
| 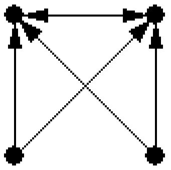 | 0.0069864% | 2.7846e-005% | 9.147e-007 | 76.076 |

| | | | | |
|---|---|---|---|---|
| 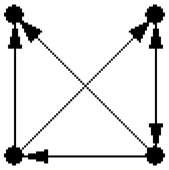 | 0.0029417% | 5.5128e-006% | 3.8842e-007 | 75.593 |
| 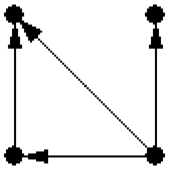 | 1.9356% | 0.13077% | 0.00024812 | 72.741 |
| 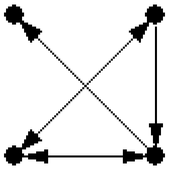 | 0.050744% | 0.00065726% | 7.0204e-006 | 71.344 |
| 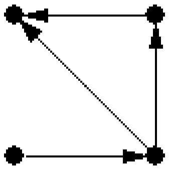 | 0.076116% | 0.0029079% | 1.063e-005 | 68.866 |
| 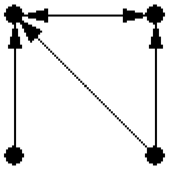 | 0.15628% | 0.0067218% | 2.2863e-005 | 65.413 |
| 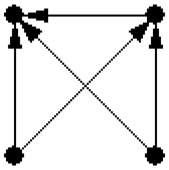 | 0.011399% | 9.0965e-005% | 1.756e-006 | 64.396 |
| 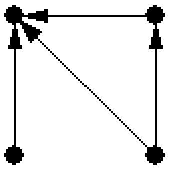 | 0.21401% | 0.014182% | 3.1287e-005 | 63.869 |
| 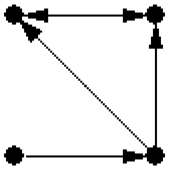 | 0.040816% | 0.00084228% | 6.3472e-006 | 62.978 |

| | | | | |
|---|---|---|---|---|
| 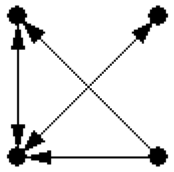 | 0.11767% | 0.0047355% | 1.8789e-005 | 60.106 |
| 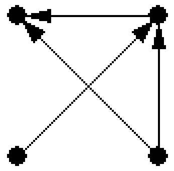 | 0.12318% | 0.0072326% | 1.9978e-005 | 58.038 |
| 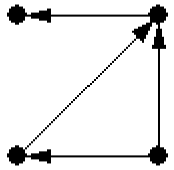 | 0.093766% | 0.0036942% | 1.5866e-005 | 56.771 |
| 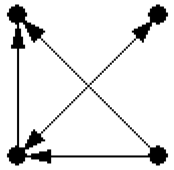 | 0.079793% | 0.0044329% | 1.4458e-005 | 52.124 |
| 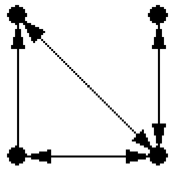 | 0.059201% | 0.0016402% | 1.1062e-005 | 52.036 |
| 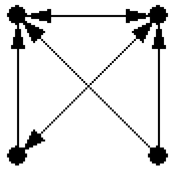 | 0.002574% | 8.8133e-006% | 5.0413e-007 | 50.882 |
| 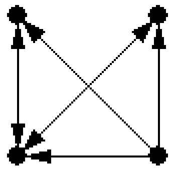 | 0.0080896% | 6.6418e-005% | 1.5795e-006 | 50.794 |
| 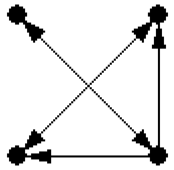 | 0.037874% | 0.0010984% | 7.2799e-006 | 50.517 |

| | | | | |
|---|---|---|---|---|
| 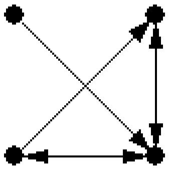 | 0.031991% | 0.00076406% | 6.1816e-006 | 50.515 |
| 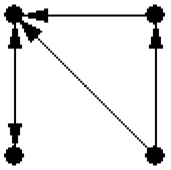 | 0.069497% | 0.0044649% | 1.4106e-005 | 46.101 |
| 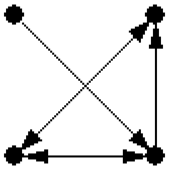 | 0.024269% | 0.00069774% | 5.2972e-006 | 44.497 |
| 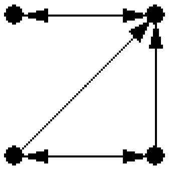 | 0.015444% | 0.00023162% | 3.4631e-006 | 43.926 |
| 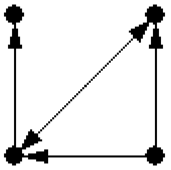 | 0.081631% | 0.0030531% | 1.8049e-005 | 43.537 |
| 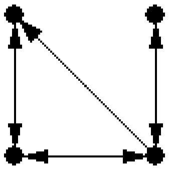 | 0.036403% | 0.0012745% | 8.1334e-006 | 43.191 |
| 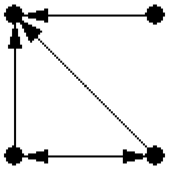 | 0.030152% | 0.00088806% | 7.043e-006 | 41.55 |
| 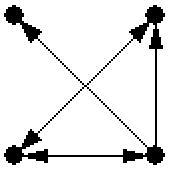 | 0.17907% | 0.0061929% | 4.2398e-005 | 40.776 |

| | | | | |
|---|---|---|---|---|
| 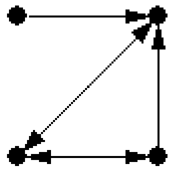 | 0.037138% | 0.0014091% | 9.3167e-006 | 38.35 |
| 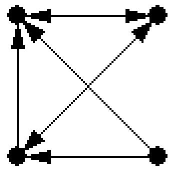 | 0.0014708% | 5.8259e-006% | 4.1049e-007 | 35.689 |
| 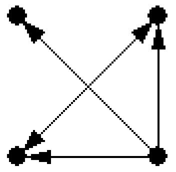 | 0.51847% | 0.038795% | 0.00014458 | 33.176 |
| 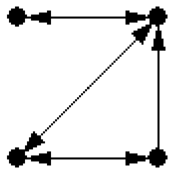 | 0.026843% | 0.0012348% | 7.9773e-006 | 32.101 |
| 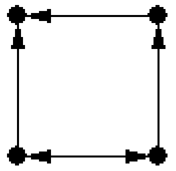 | 0.013237% | 0.00062319% | 4.0968e-006 | 30.791 |
| 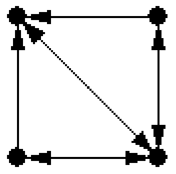 | 0.0018385% | 1.43e-005% | 6.2785e-007 | 29.055 |
| 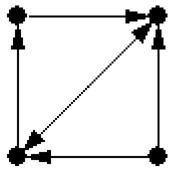 | 0.00073542% | 2.8033e-006% | 2.8033e-007 | 26.134 |
| 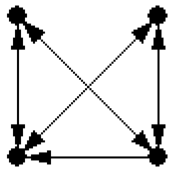 | 0.0014708% | 1.3739e-005% | 6.0313e-007 | 24.159 |

| | | | | |
|---|---|---|---|---|
| 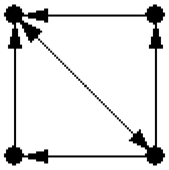 | 0.0011031% | 8.0384e-006% | 4.5942e-007 | 23.836 |
| 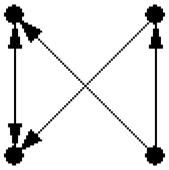 | 0.020592% | 0.0028753% | 7.9958e-006 | 22.157 |
| 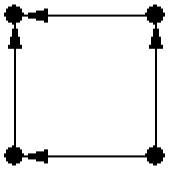 | 0.11546% | 0.023322% | 4.5161e-005 | 20.402 |
| 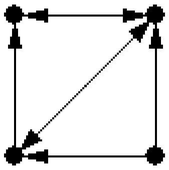 | 0.00073542% | 5.8479e-006% | 4.1142e-007 | 17.733 |
| 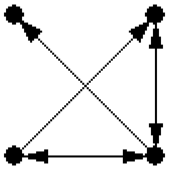 | 0.02721% | 0.00095008% | 1.4925e-005 | 17.595 |
| 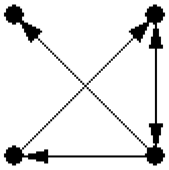 | 0.027946% | 0.0023028% | 1.5774e-005 | 16.257 |
| 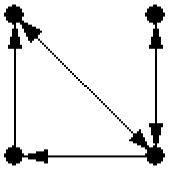 | 0.008825% | 0.00057572% | 5.2375e-006 | 15.75 |
| 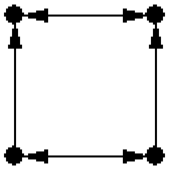 | 0.0033094% | 0.00018768% | 2.0874e-006 | 14.955 |

| | | | | |
|---|---|---|---|---|
| 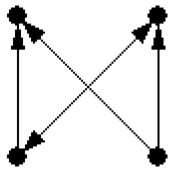 | 0.022798% | 0.0061216% | 1.1461e-005 | 14.55 |
| 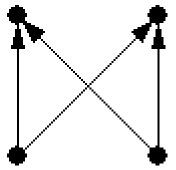 | 0.31218% | 0.1184% | 0.00013766 | 14.077 |
| 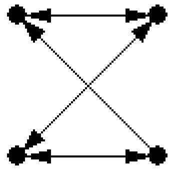 | 0.0077219% | 0.0010041% | 4.9902e-006 | 13.462 |
| 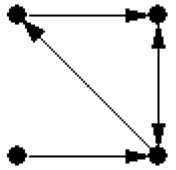 | 0.0058833% | 0.00037892% | 4.2209e-006 | 13.041 |
| 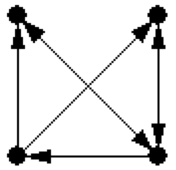 | 0.00036771% | 2.9554e-006% | 2.9554e-007 | 12.342 |
| 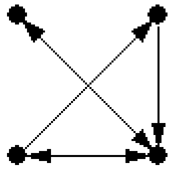 | 0.0058833% | 0.00048421% | 4.4023e-006 | 12.264 |
| 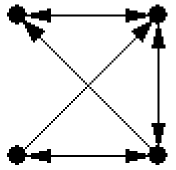 | 0.00036771% | 2.9742e-006% | 2.9742e-007 | 12.263 |
| 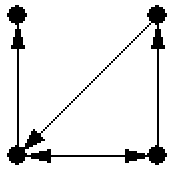 | 0.0040448% | 0.00025515% | 3.4963e-006 | 10.839 |

| | | | | |
|---|---|---|---|---|
| 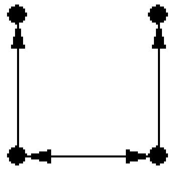 | 1.1984% | 0.52348% | 0.00070123 | 9.6242 |
| 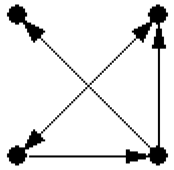 | 0.021327% | 0.0016734% | 2.0585e-005 | 9.5475 |
| 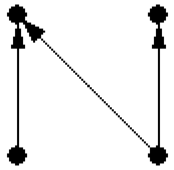 | 13.948% | 19.801% | 0.0062874 | -9.3099 |
| 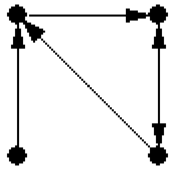 | 0.0044125% | 0.00045753% | 4.313e-006 | 9.1698 |
| 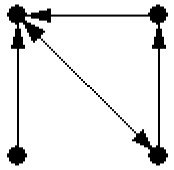 | 0.006251% | 0.00063893% | 6.1515e-006 | 9.1232 |
| 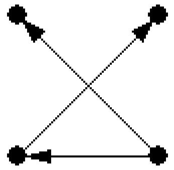 | 12.328% | 9.409% | 0.0037057 | 7.8763 |
| 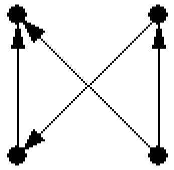 | 0.015076% | 0.0052121% | 1.2725e-005 | 7.7516 |
| 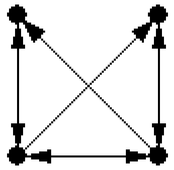 | 0.00036771% | 5.5225e-006% | 5.5225e-007 | 6.5584 |

| | | | | |
|---|---|---|---|---|
| 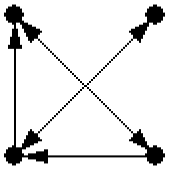 | 0.002574% | 0.00030702% | 3.5927e-006 | 6.3098 |
| 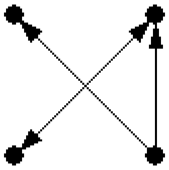 | 5.7076% | 7.6809% | 0.0033671 | -5.8608 |
| 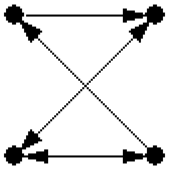 | 0.0029417% | 0.00051716% | 4.4313e-006 | 5.4714 |
| 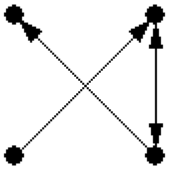 | 0.731% | 1.0919% | 0.00093059 | -3.8777 |
| 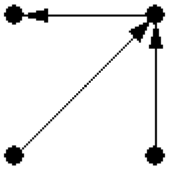 | 0.517% | 0.70708% | 0.00051109 | -3.7191 |
| 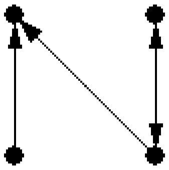 | 1.1487% | 1.6254% | 0.0014439 | -3.3013 |
| 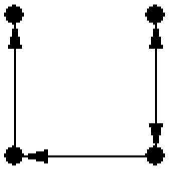 | 0.99686% | 0.7718% | 0.00074445 | 3.023 |
| 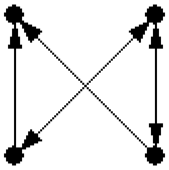 | 0.023533% | 0.011945% | 4.0168e-005 | 2.8849 |

| | | | | |
|---|---|---|---|---|
| 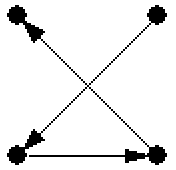 | 0.7413% | 0.96296% | 0.00079043 | -2.8043 |
| 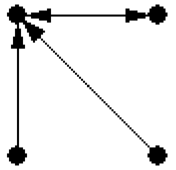 | 0.68761% | 0.85966% | 0.00063438 | -2.712 |
| 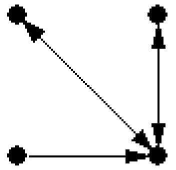 | 0.49604% | 0.62762% | 0.00057315 | -2.2958 |
| 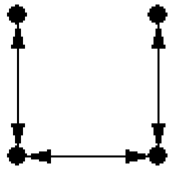 | 0.69754% | 0.88439% | 0.00093553 | -1.9973 |
| 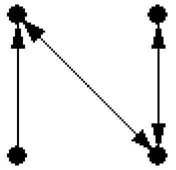 | 1.1495% | 1.395% | 0.0012568 | -1.9541 |
| 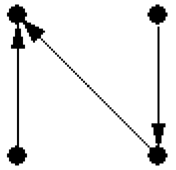 | 1.7337% | 2.0265% | 0.0015 | -1.952 |
| 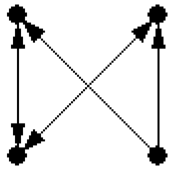 | 0.025372% | 0.019429% | 3.1035e-005 | 1.915 |
| 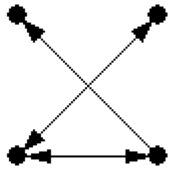 | 1.569% | 1.3247% | 0.0012769 | 1.9136 |

| | | | | |
|---|---|---|---|---|
| 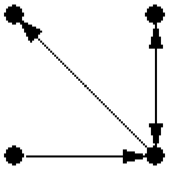 | 0.66776% | 0.78679% | 0.00064614 | -1.8423 |
| 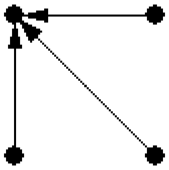 | 0.84941% | 0.98401% | 0.00074061 | -1.8175 |
| 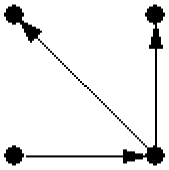 | 2.3007% | 2.6709% | 0.0021326 | -1.7357 |
| 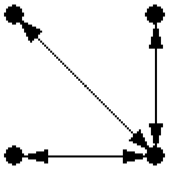 | 0.33756% | 0.42362% | 0.00051125 | -1.6835 |
| 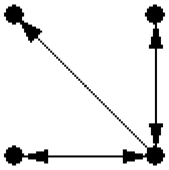 | 0.57804% | 0.6998% | 0.00084699 | -1.4376 |
| 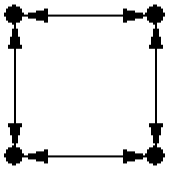 | 0.010664% | 0.0074153% | 2.3136e-005 | 1.4039 |
| 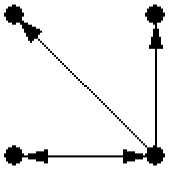 | 3.045% | 3.3621% | 0.0038197 | -0.8301 |
| 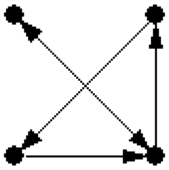 | 0.00036771% | 0.00021237% | 3.2579e-006 | 0.4768 |

| | | | | |
|---|---|---|---|---|
| 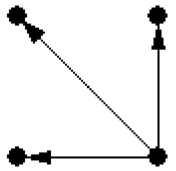 | 39.849% | 38.911% | 0.024477 | 0.38318 |
| 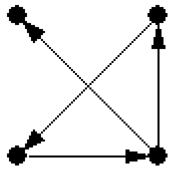 | 0.00073542% | 0.0011596% | 1.2371e-005 | -0.34289 |
| 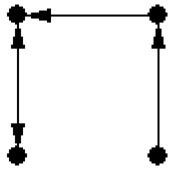 | 0.81153% | 0.79606% | 0.00062677 | 0.24679 |
| 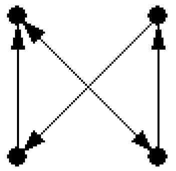 | 0.00036771% | 0.00030186% | 3.3983e-006 | 0.19377 |
| 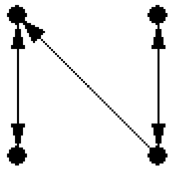 | 0.62547% | 0.63149% | 0.00064194 | -0.093734 |
| 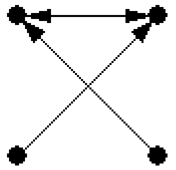 | 0.55892% | 0.5556% | 0.00045267 | 0.07325 |
| 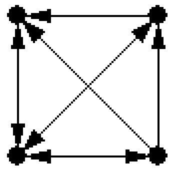 | 0.00073542% | 0% | 0 | undefined |
| 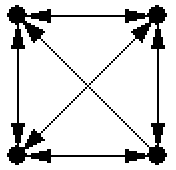 | 0.00073542% | 0% | 0 | undefined |

| | | | | |
|---|---|---|---|---|
| 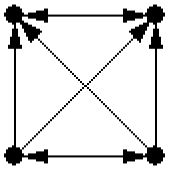 | 0.00073542% | 0% | 0 | undefined |
| 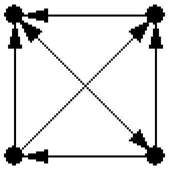 | 0.00073542% | 0% | 0 | undefined |
| 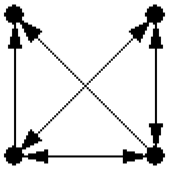 | 0.006251% | 0% | 0 | undefined |
| 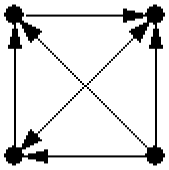 | 0.00073542% | 0% | 0 | undefined |
| 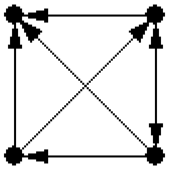 | 0.00073542% | 0% | 0 | undefined |
| 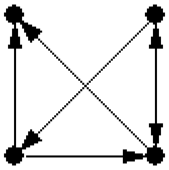 | 0.00073542% | 0% | 0 | undefined |
| 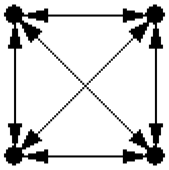 | 0.00073542% | 0% | 0 | undefined |
| 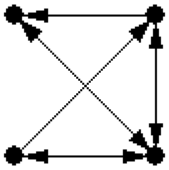 | 0.00073542% | 0% | 0 | undefined |

| | | | | |
|---|---|---|---|---|
| 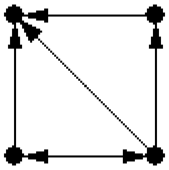 | 0.008825% | 0% | 0 | undefined |
| 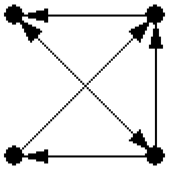 | 0.00073542% | 0% | 0 | undefined |
| 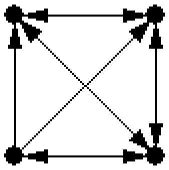 | 0.00036771% | 0% | 0 | undefined |
| 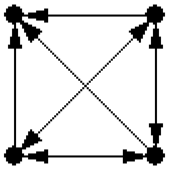 | 0.00036771% | 0% | 0 | undefined |
| 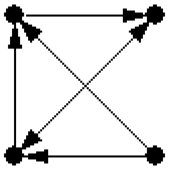 | 0.00036771% | 0% | 0 | undefined |
| 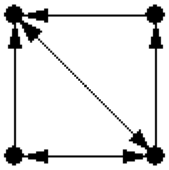 | 0.00036771% | 0% | 0 | undefined |
| 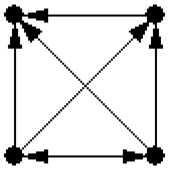 | 0.0033094% | 0% | 0 | undefined |
| 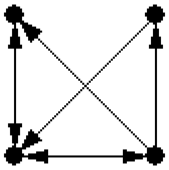 | 0.0029417% | 0% | 0 | undefined |

| | | | | |
|---|---|---|---|---|
| 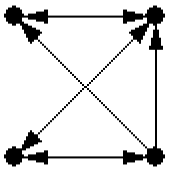 | 0.0033094% | 0% | 0 | undefined |
| 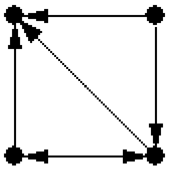 | 0.0033094% | 0% | 0 | undefined |
| 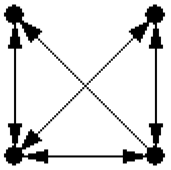 | 0.0033094% | 0% | 0 | undefined |
| 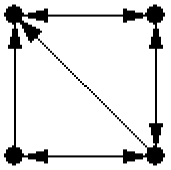 | 0.002574% | 0% | 0 | undefined |
| 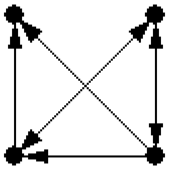 | 0.002574% | 0% | 0 | undefined |
| 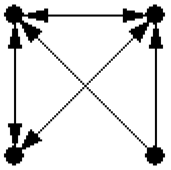 | 0.0036771% | 0% | 0 | undefined |
| 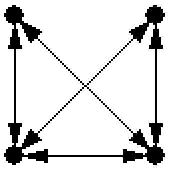 | 0.0036771% | 0% | 0 | undefined |
| 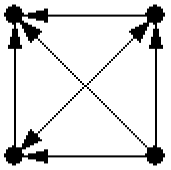 | 0.0018385% | 0% | 0 | undefined |

| | | | | |
|---|---|---|---|---|
| 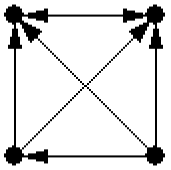 | 0.0018385% | 0% | 0 | undefined |
| 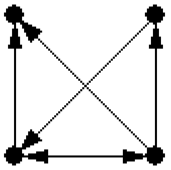 | 0.0014708% | 0% | 0 | undefined |
| 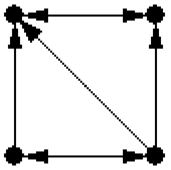 | 0.0040448% | 0% | 0 | undefined |
| 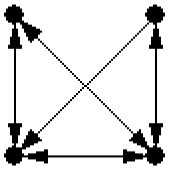 | 0.0014708% | 0% | 0 | undefined |
| 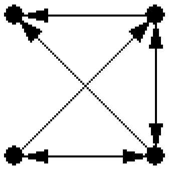 | 0.0014708% | 0% | 0 | undefined |
| 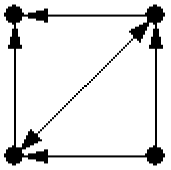 | 0.0044125% | 0% | 0 | undefined |
| 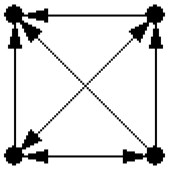 | 0.0011031% | 0% | 0 | undefined |
| 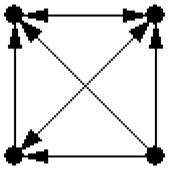 | 0.0011031% | 0% | 0 | undefined |

| | | | | |
|---|---|---|---|---|
| 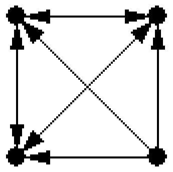 | 0.0011031% | 0% | 0 | undefined |
| 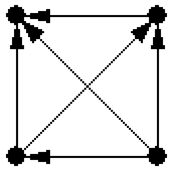 | 0.0051479% | 0% | 0 | undefined |
| 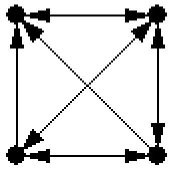 | 0.0011031% | 0% | 0 | undefined |
| 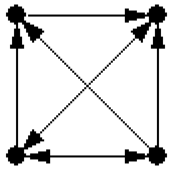 | 0.0011031% | 0% | 0 | undefined |

Supplementary Table 5: Motifs of 4 genes for the non-transcriptional model showing the abundance and the statistical significance.
